# Novel Centromeric Loci of the Wine and Beer Yeast *Dekkera bruxellensis CEN1* and *CEN2*

**DOI:** 10.1371/journal.pone.0161741

**Published:** 2016-08-25

**Authors:** Olena P. Ishchuk, Tanja Vojvoda Zeljko, Anna J. Schifferdecker, Sofia Mebrahtu Wisén, Åsa K. Hagström, Elżbieta Rozpędowska, Mikael Rørdam Andersen, Linda Hellborg, Zhihao Ling, Andrei A. Sibirny, Jure Piškur

**Affiliations:** 1 Department of Biology, Lund University, Lund, Sweden; 2 Department of Systems Biology, Technical University of Denmark, Lyngby, Denmark; 3 Department of Molecular Genetics and Biotechnology, Institute of Cell Biology, NAS of Ukraine, Lviv, Ukraine; 4 Division of Molecular Biology, Ruđer Bošković Institute, Zagreb, Croatia; 5 Department of Biotechnology and Microbiology, University of Rzeszow, Rzeszow, Poland; Saint Jude Children's Research Hospital, UNITED STATES

## Abstract

The wine and beer yeast *Dekkera bruxellensis* thrives in environments that are harsh and limiting, especially in concentrations with low oxygen and high ethanol. Its different strains’ chromosomes greatly vary in number (karyotype). This study isolates two novel centromeric loci (*CEN1* and *CEN2*), which support both the yeast’s autonomous replication and the stable maintenance of plasmids. In the sequenced genome of the *D*. *bruxellensis* strain CBS 2499, *CEN1* and *CEN2* are each present in one copy. They differ from the known “point” *CEN* elements, and their biological activity is retained within ~900–1300 bp DNA segments. *CEN1* and *CEN2* have features of both “point” and “regional” centromeres: They contain conserved DNA elements, *ARS*s, short repeats, one tRNA gene, and transposon-like elements within less than 1 kb. Our discovery of a miniature inverted-repeat transposable element (MITE) next to *CEN2* is the first report of such transposons in yeast. The transformants carrying circular plasmids with cloned *CEN1* and *CEN2* undergo a phenotypic switch: They form fluffy colonies and produce three times more biofilm. The introduction of extra copies of *CEN1* and *CEN2* promotes both genome rearrangements and ploidy shifts, with these effects mediated by homologous recombination (between circular plasmid and genome centromere copy) or by chromosome breakage when integrated. Also, the proximity of the MITE-like transposon to *CEN2* could translocate *CEN2* within the genome or cause chromosomal breaks, so promoting genome dynamics. With extra copies of *CEN1* and *CEN2*, the yeast’s enhanced capacities to rearrange its genome and to change its gene expression could increase its abilities for exploiting new and demanding niches.

## Introduction

On each chromosome, the centromere (*CEN*) is the essential and specialized locus at which kinetochore is formed. As the site of spindle attachment, it is responsible for the proper segregation (stable maintenance) of its chromosome to its daughter cells. All centromeres are associated with the centromeric histone H3 (CenH3) [1, 2].

Paradoxically, while the chromosome segregation machinery on the centromere is conserved in all eukaryotes, the centromere is among the fastest evolving parts of each chromosome [3, 4]. While its function is shared by all species, centromeres have structures that vary between species, and even within the chromosome set of particular species [1, 5–7].

Two centromere subtypes are “point” and “regional”. Simple “point” centromeres (~200 bp long) have their function determined by their DNA sequences; they are found on budding yeasts. When “point” centromeres are cloned on plasmids, they stabilise the plasmids during cell division, so that the stabilised plasmids act as mini-chromosomes [8–10]. Other eukaryotes possess complex “regional” centromeres (~40 kb–several Mb) that consist of arrays of repetitive DNA (tandem repeats, direct and inverted repeats, transposable elements); their functions are determined epigenetically [6, 11–14]. The transposable elements (TEs) accelerate the evolution of eukaryotic genomes [15]. Spread widely through the genome, TEs are often found near “regional” centromeres and accelerate the evolution of centromeres [16].

**Fig 1:**
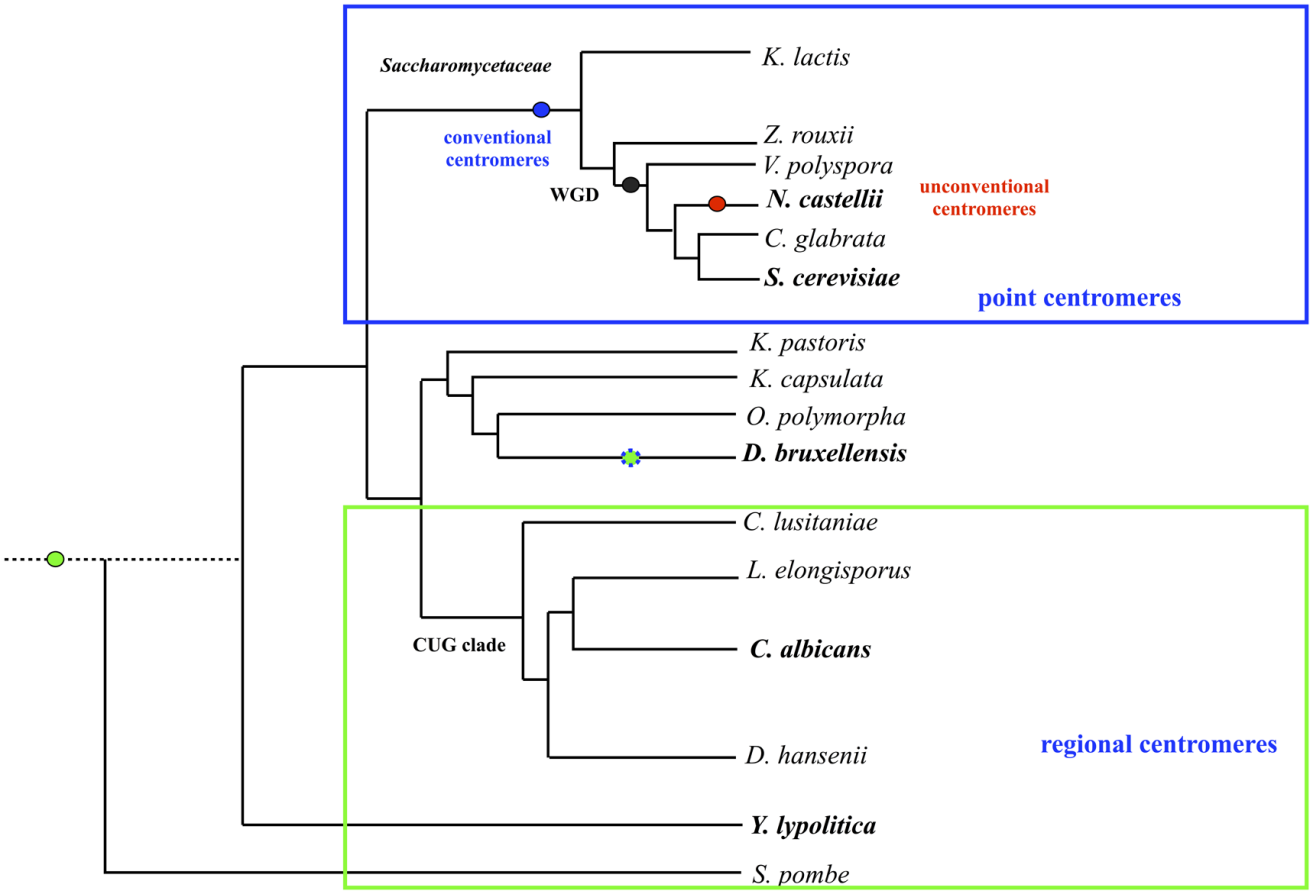
Yeast phylogeny and centromeres. The centromeres are signified by coloured ovals. Our cloned *D*. *bruxellensis* centromeres have features of both “regional” and “point” centromeres, and they appear in *green—blue*. The “conventional point” centromeres appear in *blue*; the “unconventional point” centromeres in *red*; the “regional” centromeres in *green*. A black oval displays the whole-genome duplication event (WGD). The phylogeny is adapted from Curtin and Pretorius, 2014, and the phylogeny and centromeres are adapted from Kobayashi et al., 2015.

The whole-genome duplication (WGD) yeasts have two types of “point” centromeres: “conventional” centromeres and “unconventional” centromeres (Fig 1). The baker’s yeast *Saccharomyces cerevisiae* has “conventional” centromeres: its minimal functional *CEN* DNA is 125 bp long, is conserved between all chromosomes, and consists of the three centromere DNA elements (CDEs): CDEI, CDEII and CDEIII [14, 17]. The yeast *Naumovozyma castelii* has “unconventional” centromeres, which contains three CDEs (CDEI, CDEII, and CDEIII) with new consensus motifs [18].

The centromere DNA of the pre-WGD yeast *Dekkera bruxellensis* has not been studied in a scientific publication. The yeast *D*. *bruxellensis* has industrial importance and it is a model organism of evolution. While it is one of the main spoilage organisms in the wine and bioethanol industry, it contributes flavours to Belgium lambic and gueuze beer and to some red wines [19]. *D*. *bruxellensis* shares with *S*. *cerevisiae* several abilities: high ethanol production and tolerance, an ability to produce ethanol in the presence of oxygen and excess glucose (Crabtree-positive), an ability to grow under anaerobic conditions, and a generation of viable, respiratory deficient, petite mutants [20, 21]. However, these abilities have developed independently by parallel evolution since the *D*. *bruxellensis* and *S*. *cerevisiae* lineages diverged at least 200 million years ago (mya) from a yeast progenitor (Fig 1) that was a poor ethanol-producer and that needed aerobic conditions for propagation [21, 22]. Comparative genomics classified this yeast species as a sister group to the methylotrophic yeasts *Pichia* (*Komagataella*) *pastoris* and *Ogataea polymorpha* [23, 24], which are aerobic and Crabtree-negative. While *D*. *bruxellensis* shares with methylotrophic yeasts some metabolic features (such as nitrate assimilation [22, 25, 26]), *D*. *bruxellensis*’s chromosomes differ in number [27], unlike methylotrophic yeasts or baker’s yeast. Besides having extreme karyotype variability, the genome of *D*. *bruxellensis* also contains heterozygous chromosome sets [23, 24, 28]. The karyotype diversity of *D*. *bruxellensis* is evidence of its a dynamic genome and in particular of its chromosomes having been repeatedly reshaped, we note. Similar genome flexibility occurs in the *Candida* yeasts, which reshape their genomes to survive in hosts [1, 29–31]. For humans, aneuploidy and karyotype changes are associated with pathologies like cancer.

*The mechanisms underlying D*. *bruxellensis karyotype variability and genome dynamics are unknown*. The current study focused on the cloning of the yeast’s centromere regions, because of their suspected role in chromosome reshaping. From our newly created gene library, we discovered two novel centromeric loci (*CEN1* and *CEN2*), which display features of both “point” and “regional” centromeres. Within less than 1 kb, *CEN2*’s neighbourhood contains a miniature inverted-repeat transposable element (MITE). MITE-transposons had been reported in plants and animals but not in yeast. Experiments showed that *CEN1* and *CEN2* were associated with changing karyotype, with ploidy shifts, and with the increase of biofilm formation. This experimental evidence and our analysis of the structure and properties of *CEN1* and *CEN2* suggest that these loci could aid in adaptation of *D*. *bruxellensis* to new environments, for example, nutrient limitation.

## Materials and Methods

### Strains and growth conditions

The yeast strains (Table 1) were grown at 25°C in complete medium YPD (0.5% yeast extract, 1% peptone, 2% glucose) or minimal medium YNB (yeast nitrogen base without amino acids and ammonium sulphate) supplemented with 0.5% ammonium sulphate and 2% glucose. For the *ura3* mutant Y997 [32], 50 mg/l uracil was added to the YNB medium. Prototroph transformants of Y997 mutant were selected on the minimal medium YNB.

**Table 1:** Yeast strains used in this study.

Laboratory designation	Original strain designation	Description, reference
Y601	*S*. *cerevisiae* Hansen ATCC 204517	MATalpha *ura3 ade1 leu1 his2 trp1 met14 gal1*
Y865	*D*. *bruxellensis* CBS 96	Wild-type
Y879	*D*. *bruxellensis* CBS 2499	Wild-type
Y880	*D*. *bruxellensis* CBS 2547	Wild-type
Y881	*D*. *bruxellensis* CBS 2796	Wild-type
Y883	*D*. *bruxellensis* CBS 3025	Wild-type
Y891	*D*. *bruxellensis* CBS 4602	Wild-type
Y900	*D*. *bruxellensis* CBS 4481	Wild-type
Y901	*D*. *bruxellensis* CBS 4482	Wild-type
Y997	-	*ura3*, [32]
Y1376	*Kluyveromyces lactis* CBS 2359	Wild-type
Y1377	-	Transformant of Y997 strain carrying one copy of SacI fragment of P935 integrated into the genome

For a bacterial host, experiments used the *Escherichia coli* strain XL1-Blue (Stratagene), which was grown at 37°C in Luria-Bertani (LB) [33]. The transformed *E*. *coli* cells were maintained on a LB medium containing 100 mg/l of ampicillin.

### Gene library: Construction and screening

For the cloning of centromeres, our gene library was constructed following a protocol for *S*. *cerevisiae* [34]. The *D*. *bruxellensis* strain Y881 served as a source of genomic DNA. High molecular-weight total DNA was extracted from spheroplasts [35]. The gene library was constructed on the basis of the plasmid P892, which carries *URA3* gene as a selective marker [32]. The *D*. *bruxellensis* genomic DNA was partially digested with *Sau*3AI, after which 3–10 kb *Sau*3AI fragments were sub-cloned into the *Bam*HI site of P892; the resulting plasmids were transformed into the electrocompetent *E*. *coli* XL1-Blue strain. Then PCR analysis estimated that 60% of the library’s recombinant plasmids contained an insert. The final library was prepared from roughly 200,000 bacterial transformants (with the 200 thousand being 10 times the genome coverage); it was used to transform the *D*. *bruxellensis* Y997 *ura3* mutant. The yeast Ura^+^ transformants were selected on the minimal medium YNB. The gene-library plasmids were isolated from Y997 Ura^+^ transformants through the transformation of *E*. *coli* with the total DNA of the yeast transformant. Since the gene library vector carried both an ampicillin-resistance gene and the bacterial *ori* sequence, the plasmids were purified from bacterial transformants and further analysed by sequencing.

The oligonucleotides used in this study are listed in S1 Table. Plasmids constructed in this study are listed in Table 2 and in S1 Materials and Methods.

**Table 2:** Plasmids used in this study.

Plasmid, laboratory designation	Description	Reference
P891	pDb2URA3, plasmid carrying the *URA3* gene of *D*. *bruxellensis*, *ARS/CEN* of *S*. *cerevisiae* (from pYC130)	This study
P892	*D*. *bruxellensis* integrative plasmid carrying *URA3* of *D*. *bruxellensis*	[32]
P893	pDbpURA3, 2-micron plasmid of pYES2 origin carrying *URA3* of *S*. *cerevisiae*, *S*. *cerevisiae GAL1* promoter is replaced by *D*. *bruxellensis* promoter *URA3*	This study
P935	*D*. *bruxellensis* integrative reporter plasmid carrying *LAC4* of *K*. *lactis* under the *D*. *bruxellensis YPR100W* (*MRPL51*, Mitochondrial ribosomal protein of the large subunit) promoter	This study
P1160	*D*. *bruxellensis* replicative reporter plasmid carrying *CEN2* (1–2407 bp) of Y879 strain, *LAC4* of *K*. *lactis* under the *D*. *bruxellensis YPR100W* (*MRPL51*, Mitochondrial ribosomal protein of the large subunit) promoter	This study
P1211	*D*. *bruxellensis* replicative reporter plasmid carrying *CEN2* (1–2602 bp) from P950, *LAC4* of *K*. *lactis* under the *D*. *bruxellensis YPR100W* (*MRPL51*, Mitochondrial ribosomal protein of the large subunit) promoter	This study
P949	gene library origin, 3276 bp insert size (61.02% of AT-content), carrying *CEN1* (0.916 kb)	This study
P947	gene library origin, 3252 bp insert size (61.13% of AT-content), carrying *CEN1* (0.916 kb)	This study
P1172	P892 carrying *CEN1* of Y881 strain (1566–2482 bp), sub-cloned fragment originating from gene library plasmid P949	This study
P950	gene library origin, 2602 bp insert size (63.23% of AT-content), *CEN2* of Y881 strain	This study
P1210	P892 carrying full length *CEN2* of Y879 (1–2407 bp)	This study
P1202	P892 carrying *CEN2-1* (1–1921 bp)	This study
P1173	P892 carrying *CEN2-2* (1925–2120 bp)	This study
P1174	P892 carrying *CEN2-3* (2121–2602 bp)	This study
P1209	P892 carrying *CEN2-4* (1925–2602 bp)	This study
P1038	P892 carrying *CEN2-5* (1290–2602 bp)	This study

### Analysis of plasmid stability

The plasmid stability in Y997 Ura^+^ transformants was studied by alternative cultivation in the non-selective medium YPD and in the selective medium YNB. A single colony was inoculated into liquid YPD and grown for 20 generations. The cultures were diluted and plated on YPD plates following the growth at time points of 10 and 20 generations. The colonies obtained on YPD plates after 3 days were replicated on the minimal medium YNB. The plasmid stability was then estimated by the percentage of the colonies that remained prototrophic on the selective medium. For each plasmid, plasmid loss was calculated per generation. Each transformant selected for analysis was confirmed to have autonomous plasmid status by the re-isolation of the plasmid through bacteria (to rule out the occurrence of rare random integration-events).

### Biofilm and mat formation assay

For the biofilm assay, the yeast strains were grown to their stationary phases in the minimal medium with 2% glucose. Then they were transferred to the YNB (supplemented with 0.2% glucose pH 7.0) at the final concentration 0.2 OD/ml, where they were incubated for 48 hours at 30°C. Their biofilm was measured by crystal-violet staining [36]. The yeast mats were grown in the YPD medium containing 0.3% agar at 25°C for 30 days.

### DNA content analysis by flow cytometry

The samples were prepared for flow cytometry using a protocol for fission yeast [37]. The mid-log cultures of the *D*. *bruxellensis* grown in YNB with glucose were washed with sterile water and then fixed with 70% ethanol. The DNA was stained by SYTOX green (S7020, Invitrogen, Life Technologies). The fluorescence measurements from 20,000 cells were collected using Beckman Coulter Moflo XDP and analyzed by FlOWJO software.

### Chromatin co-immunoprecipitation

The chromatin co-immunoprecipitation (ChIP) was performed using standard protocols [2, 38]. The early logarithmic cultures (with OD_600_ ~1.0) were harvested, were washed with water, and were fixed in 1% formaldehyde for 30 min at 25°C. The reaction was stopped by incubation with 125 mM glycine on ice for 15 min. The cells were subsequently pelleted at +4°C at 200g for 10 min and washed once with the PBS buffer having pH 7.4. To obtain spheroplasts, the cells were treated with 0.5 mg/ml of zymolyase (US Biological, Cat. no. Z1004) with the addition of the protease-inhibitor cocktail cOmplete^™^ (Roche, Cat. no. 04693159001). The spheroplasts were lysed for 30 min on ice in the lysis buffer (25 mM HEPES/KOH pH 8.0, 50 mM KCl, 10 mM MgSO4, 10 mM Na citrate, 25 mM Na sulphite, 0.25% TritonX-100, 3 mM DTT, and 100 μg/ml of RNAse A); they were sonicated to yield DNA fragments of 500–3000 bp. After the cell-debris had been precipitated by centrifugation at +4°C at 20 817 g for 15 min, the input lysate was collected. This lysate was incubated at +4°C overnight in two conditions: with or without the 4 μg/ml antibodies for the *C*. *albicans* centromeric histone H3 (raised for N-terminal protein part, 1–18 aa), which had been kindly provided by Judith Berman. The next day, the antibody and its associated DNA were coupled to the Dynabeads Protein A/G magnetic beads using the MAGnify Chromatin Immunoprecipitation system (Invitrogen, Cat. no. 49–2024); the resulting beads were repeatedly washed with subsequent elution and crosslinks reversion following Invitrogen’s instructions. The purified DNA was used for standard procedures, including PCR analysis, Southern blotting, and sequencing.

### Southern blotting of transformants and chromosomes

For the *D*. *bruxellensis* transformants 10 μg of their total DNA was digested with restriction enzymes (either *Eco*RI or *Bgl*II) and then separated on 0.8% agarose gel.

For the strains of *D*. *bruxellensis*, their chromosome-sized DNA was prepared (following [39]), after which their chromosomes were separated by pulse-field gel electrophoresis (PFGE) using a CHEF Mapper XA (Bio-Rad) and the following 4-step programme: (1) a 2700s pulse for 25 h, at 1.5 V/cm and the angle of 53°; (2) a 2200s pulse for 25 h, at 1.5 V/cm and the angle of 60°; (3) a 1500s pulse for 30 h, at 2 V/cm and the angle of 60°; (4) a 500s pulse for 30 h, at 2.5 V and the angle of 60°.

The separated digested DNA or chromosomes were transferred onto the Hybond XL-membrane and hybridized with radioactively labeled DNA probes. The DNA labelling was performed with [γ-^32^P] dCTP using the Amersham Rediprime II Random Prime labelling system (GE Healthcare, Freiburg, Germany). The signals were detected using the Imaging Screen-K (35×43 cm; Bio-Rad) and Personal Molecular Imager FX (Bio-Rad).

### The plasmid copy number: Estimation by qRT-PCR

From the transformants carrying the *D*. *bruxellensis CEN1* and *CEN2* plasmids, the total DNA was used in equal amounts as templates in qRT-PCR reactions, which used the SYBR GreenER qPCR SuperMix from Invitrogen (Cat. no. 11762–500). The reactions used the primers for the genes *URA3* (URA3-1 and URA3-2) and the *YML085C* gene encoding α-tubulin (ORF085-1 and ORF085-2), as shown in S1 Table. The PCRs were run in duplicate and from two independent transformants in a RotorGene 2000. The take-off (Ct) values were used to estimate the relative gene copy-number. The recipient strain had one endogenous *URA3* gene copy; its transformant, the Y1377 strain, which carried an additional *URA3* gene because it contained the *Sac*I fragment of the P935 plasmid (Table 2). This recipient strain and its tranformant were used as controls to estimate the relative gene copy-number of the *URA3* gene. The *YML085C* gene served as an endogenous control, and its Ct measurements were used to normalize each sample.

### Sequencing and analysis of centromeres

The sequencing of the plasmids was performed by MWG Biotech (Ebersberg, Germany). The sequences were analysed, aligned, and compared by using Geneious^®^ 9.0.4 and BioEdit (http://www.mbio.ncsu.edu/BioEdit/bioedit.html). Homologous sequences were searched for in two NCBI bioinformatics databases, the Nucleotide collection and the whole-genome shotgun (WGS) contigs database, using the blastn and tblastx algorithms (http://blast.ncbi.nlm.nih.gov/Blast.cgi). For autonomously replicating sequences (*ARS*s), for CDEI-like and CDEIII-like elements (allowing 1 bp mismatch), and for novel motifs (specific to *D*. *bruxellensis*), their presence was tested using custom scripts. Repetitive elements were found by GIRI (Genetic Information Research Institute, http://www.girinst.org) and EMBOSS explorer (http://emboss.bioinformatics.nl/cgi-bin/emboss/palindrome). We searched for the tRNA gene sequences in the transfer RNA database (http://trna.bioinf.uni-leipzig.de/DataOutput/Search).

To determine the frequency of appearance, the sequences of cloned centromeres were searched against the assembled scaffolds database (repeats masked) of the *D*. *bruxellensis* CBS 2499 (Y879) genome using blastn [24]. The E-value < 1e-10 was set as a threshold to filter the blast results, and only the filtered hits (with aligned length longer than one third of the total length of the query) were kept and used for analysis.

## Results

### Cloning of novel centromeres: *CEN1* and *CEN2*

For the yeast *Dekkara bruxellensis*, *the scientific literature has lacked reports of replication plasmids or descriptions of its centromeres*.

In our study, the circular plasmids carrying an autonomously replicating sequence (*ARS*), a centromere, or the 2-micron ori loci of *Saccharomyces cerevisiae* (plasmids P891 and P893, Table 2) resulted in no *D*. *bruxellensis* transformants, likely because the replication loci of *D*. *bruxellensis* and *S*. *cerevisiae* differ.

In many (and perhaps most) budding yeasts, the centromere is functional when it is cloned on the circular plasmid [8, 9]. To clone centromere elements from the *D*. *bruxellensis* genome, we constructed a genomic library from the Y881 strain (Materials and Methods), which is the parental strain of the *ura3* mutant used (Table 1). The plasmid P892 (gene library vector backbone) did not generate any transformants of the *ura3* mutant in a circular state. Our gene library carried random genomic inserts of approximately 3–10 kb, and our library’s transformation resulted in several prototrophic transformants of the *ura3* strain. The plasmids isolated from these transformants were used for the second-round yeast transformation to investigate their properties.

**Fig 2:**
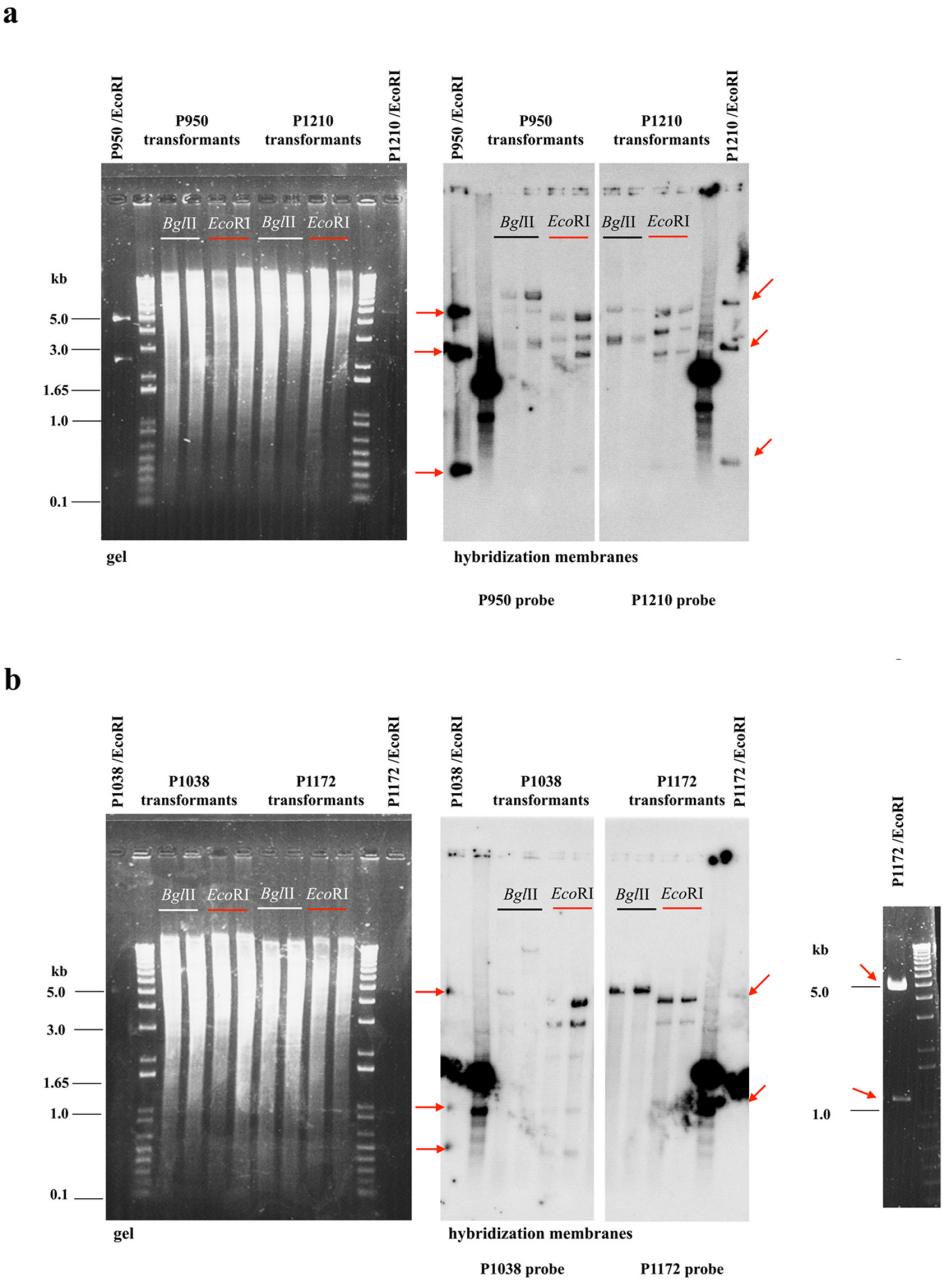
Southern hybridization of the total DNA of the transformants carrying *CEN1* or *CEN2* plasmids. (a) The gel and hybridization membrane for the *CEN2* plasmid transformants P950 and P1210. (b) The gel and hybridization membrane obtained for the *CEN2-5* (P1038) and *CEN1* (P1172) transformants. The total DNA was digested either with *Eco*RI (present on the plasmids) or *Bgl*II (absent on the plasmids). Digested with *Eco*RI, the control plasmids were P950 (*CEN2* of Y881), P1210 (*CEN2* of Y879), P1038 (*CEN2-5* deletion plasmid of *CEN2*) and P1172 (*CEN1*). Linearized by *Pst*I (P950, P1210 and P1038) or *Hind*III (P1172) and labeled with [γ-^32^P] dCTP, the whole plasmid was used as the hybridization probe. In the figure, the *Eco*RI restriction-fragments of the corresponding plasmids are shown with arrows. As all the plasmids have a pUC57 segment, the probes also hybridized with 1 kb ladder (10787018, Invitrogen, Life Technologies).

**Fig 3:**
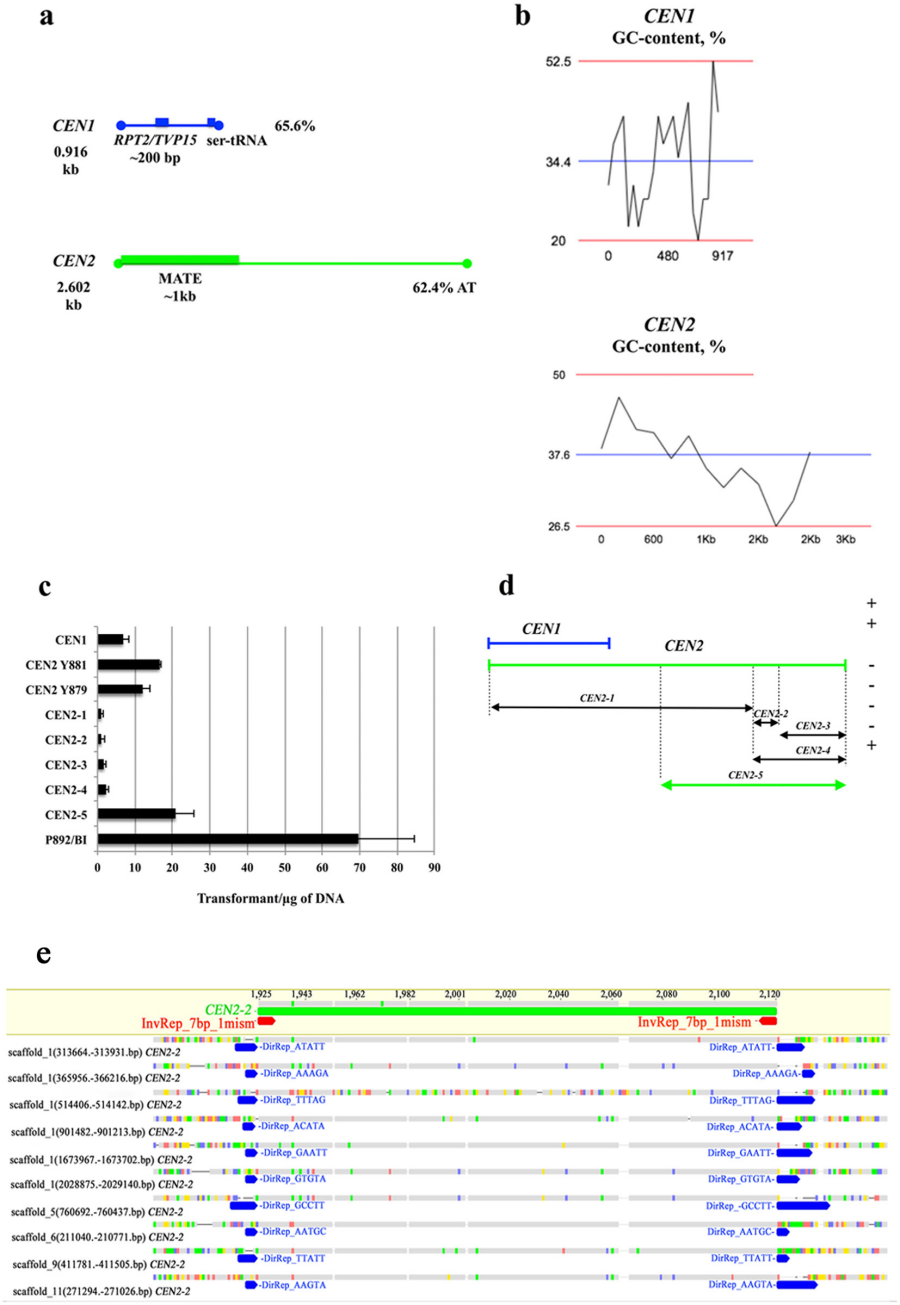
The analysis of *CEN1* and *CEN2* properties. (a) The *CEN1* locus has 65.6% AT content and carries the ser-tRNA gene; it carries part of a gene homologous to the *RPT2* gene, the *TVP15* gene, and the *COPI*-coated vesicle-protein encoding gene. The *CEN2* locus has 62.4% AT content and it carries the gene coding for the MATE efflux family protein. (b) The AT-peaks occur at the intergenic region in *CEN1* and within a 3’-region of *CEN2*. This subfigure was drawn using the DNA/RNA GC Content Calculator (http://www.endmemo.com/bio/gc.php). (c) The transformation efficiency of three plasmid types. (1) The plasmid with *CEN1*. (2) Two plasmids with *CEN2*, specifically the Y881 strain possessing the transposon and the Y879 strain lacking the transposon. (3) The deletion plasmids of *CEN2*, specifically *CEN2-1*, *CEN2-2*, *CEN2-3*, *CEN2-4*, *CEN2-5*; the plasmid *CEN2-2* has a transposon. The P892/BI plasmid, which carried the *URA3* gene and which had been linearized by *Bam*HI (BI), was used as a control. (d) The schematic representation of the *CEN1*, *CEN2* and *CEN2* deletion-plasmids. The sequences that promote autonomous replication and stable maintenance are marked with a plus sign (+); the remaining sequences are marked with a minus sign (-). (e) The MITE-like transposon found within the *CEN2* fragment (from 1925 to 2120 bp), named *CEN2-2*, was used as a query in a search through the *D*. *bruxellensis* genome (Y879 strain). 10 sequences (and their surroundings) were randomly sampled from the 51 present in the genome (S2 Table) and aligned with *CEN2-2* (reference sequence) to present the “structural” parts of the transposon: inverted and direct repeats. The bases identical to the reference sequence are shown in grey. Inverted repeats are 7 bp long and they are at the beginning and end of the transposon; marked with red arrows only on the reference sequence, inverted repeats are present in all sequences in the genome. Direct repeats are marked with blue arrows; they are 5 bp long but have different sequences, and they immediately flank the transposon (in the genome of Y879 strain).

Although several plasmids were isolated from the Y881 strain gene-library, our sequencing and genome analysis showed that they had two common inserts, which we designated *CEN1* and *CEN2* (Table 2). The insert *CEN1* was located within a bigger fragment, which had resulted from the ligation of loci on the plasmids P947 and P949; the larger fragment was then sub-cloned to a single locus on the P892 vector, resulting in the new plasmid P1172. The plasmids P1172 and P950 (having the *CEN1* and *CEN2* loci respectively) were shuttled from *D*. *bruxellensis* through the bacterium *E*. *coli*. The native forms and their restriction pattern remained unaltered in yeast cells (P950 and P1172, S1 Fig). After Southern hybridization with plasmid probes, the total DNA (which had been isolated from the yeast transformants) had the restriction pattern of the native plasmids that had been digested with the *Eco*RI enzyme (Fig 2a and 2b). Because the plasmids *CEN1* and *CEN2* do not integrate into the *D*. *bruxellensis* genome, they autonomously replicate. The transformation efficiency of each of the replicative *CEN1* and *CEN2* plasmids was less than the efficiency of the linearized plasmid P892 (Fig 3c): thus the *CEN1* and *CEN2* plasmids contain no strong *ARS* elements.

To study the stability of the plasmids, the transformed cells were propagated in non-selective media (YPD) and in selective media (YNB), as discussed in Materials and methods; Table 3. For the *D*. *bruxellensis* strains Y879 and Y997, when cells were transferred from liquid cultures to the YNB and YPD media, more YPD colonies survived and 20–30% of the YNB colonies died. To avoid such mortality, we obtained single colonies by taking two samples from the liquid YPD medium after 10 and 20 generations and plating each on solid YPD plates. Although cells stayed under non-selective conditions longer than 10 and 20 generations, two samples separated by 10 generations provided sufficient time for the calculation of plasmid loss. The plasmid loss per generation ranged from 0.4% to 2.0% (Table 3). Growing transformants for longer periods under non-selective conditions, e.g., 96 hours in YPD (approximately 32 generations) did not result in complete plasmid loss. This indicates that the plasmid loss was lower than 5% per generation, a rate in the range of stability for plasmids that carry centromeres.

**Table 3:** *CEN1* and *CEN2* plasmids stability in Y997 transformants.

$	Motif	Stability after 10 generations^#^ in liquid YPD (%)	Stability after 20 generations^#^ in liquid YPD (%)	Plasmid loss per generation (%)
P950	*CEN2*	40	20	2.0
		25	21	0.4
		39	25	1.4
P1172	*CEN1*	23	18	0.5
		44	40	0.4
		36	28	0.8
P1038	*CEN2-5*	28	13	1.5
		15	5	1.0
		25	15	1.0
P892/*Bam*HI*	None	100	100	0.0
		100	100	0.0
		100	100	0.0

^#^—After the displayed number of generations in liquid YPD, cultures were plated on solid YPD plates to obtain single colonies. After 3–4 days of growth on solid YPD plates, colonies were replicated onto solid YNB plates to calculate plasmid loss.

*—The strain carrying the plasmid P892 was linearized with *Bam*HI and integrated into the genome was used as a control.

### Sequence analysis of plasmid inserts from gene library

The plasmids isolated from our Y881 (CBS 2796) gene library were sequenced, and their sequences were aligned with the genome sequence of *D*. *bruxellensis* Y879 strain (CBS 2499), using both the whole genome shotgun (WGS) contig database and the DOE Joint Genome Institute (JGI) genome database [24]. The alignment results revealed the presence of two genome loci (identified within the inserts of the P947, P949 and P950 plasmids, Table 2). These two fragments were rich in AT base-pairs and they were collinear with the *D*. *bruxellensis* genome. As discussed in the previous section, these fragments were shown to promote stable plasmid maintenance and other centromere functions, and so we designated these centromeric loci as *CEN1* (fragment 1566–2482 bp of KF268952, Genbank) and *CEN2* (Genbank KF268953), as shown in Fig 3a.

The *CEN1* locus, which was 917 bp long, had average AT-content of 65.6% with AT-peaks between 0–480 bp and 480–917 bp (Fig 3b). In the WGS contig database, the full length of this locus aligned with *D*. *bruxellensis* CBS 2499 DEKBRscaffold_4 contig 453. The part from 1 to 779 bp aligned to *D*. *bruxellensis* LAMAP2480 contig01469. The part of *CEN1* (830–901 bp) carried the ser-tRNA gene, which gave a few hits in the genomes of the *D*. *bruxellensis* strains CBS 2499, LAMAP2480, and AWRI1499. In the JGI database for the *D*. *bruxellensis* strain CBS 2499, the *CEN1* fragment aligned with Dekbr2|scaffold_4:947305–953253. In the NCBI gene bank, ~200 bp of *CEN1* aligned with a few genes coding for the COPI-coated vesicle proteins and with the genes *RPT2* and *TVP15*. The chromosome loci flanking these genes were analysed using the sequenced genomes of *S*. *cerevisiae* and *Candida albicans*. In *S*. *cerevisiae* S288C, the *RPT2* gene is located next to the ser-tRNA gene and both genes are within ~10 kb of the centromere region *CEN4* (http://www.yeastgenome.org; http://ygob.ucd.ie). In *C*. *albicans* SC5314, the COPI-coated vesicle proteins homologue, *C7_01950W_A* gene, is located within 7 kb of *CEN7* (http://www.candidagenome.org).

The *CEN1* locus was amplified using the primers OP98 and OP99 from all of the seven *D*. *bruxellensis* strains that were tested: Y879, Y881, Y891, Y880, Y865, Y883 and Y900 (Table 1; S2 Fig).

The *CEN2*, which was 2602 bp long, had the mean AT-content of 63% with an AT-peak between 2.0–2.6 bp (Fig 3b). In the WGS contigs database, the full length of this locus, except for a short region of 1925–2120 bp (designated *CEN2-2*), aligned with *D*. *bruxellensis* CBS 2499 DEKBRscaffold_6_Cont493. This region is present in *D*. *bruxellensis* CBS 2499 in 51 copies in different genomic loci (S2 Table), is flanked by inverted repeats and by short 5 bp direct repeats (Fig 3e); consequently, it was classified as a transposon (Fig 3e). The size of the found transposon (196 bp) is consistent with the size of the miniature inverted-repeat transposable elements (MITEs) reported in the genomes of plants and animals [40, 41]. In the NCBI gene bank, *CEN2* partially (within the first 1000 bp with the highest GC content) aligned to sequences from different yeast species and to filamentous fungi coding for protein and to genes coding for the putative MATE family drug/sodium antiporter.

The *CEN1* and *CEN2* loci had one copy in the genome of Y879 (S2 Table). The *CEN1* part, where ser-tRNA gene was located, gave hits to other scaffolds and the transposon part of *CEN2* (designated *CEN2-2*) gave 51 hits (S2 Table).

**Fig 4:**
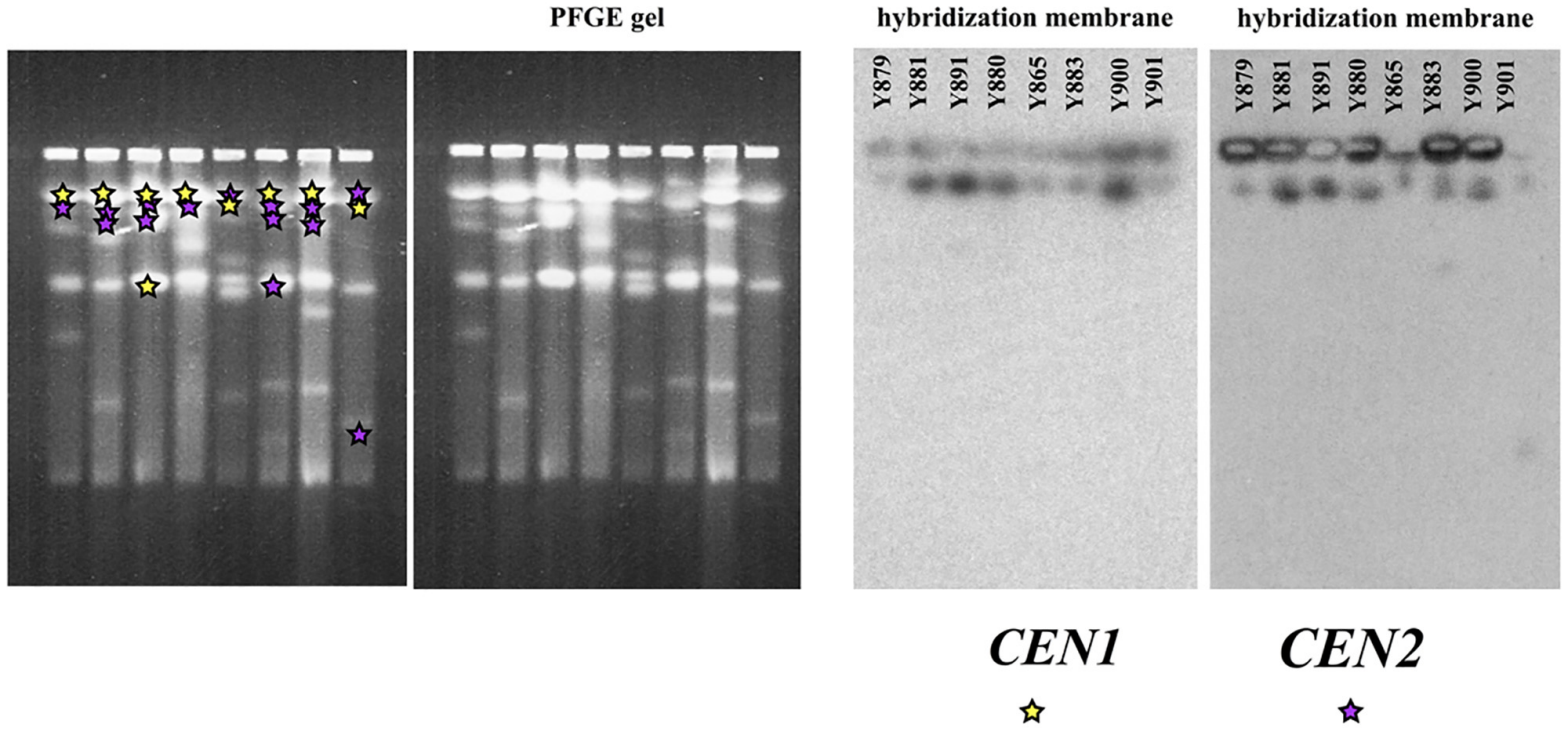
Hybridization of *CEN1* and *CEN2* to chromosomes. The chromosome-sized DNA of the strains (Y879, Y881, Y891, Y880, Y865, Y883, Y900, and Y901) was separated on PFGE gel and hybridized with *CEN* probes. For the *CEN1* locus, the full fragment was used as a probe (amplified with primers OP98 and OP99). For the *CEN2* locus, the *CEN2-1* part (amplified with primers OP91 and OP127) was used as a probe; this was performed to avoid the transposon part, which is present in the genome in several copies. The hybridization signals for both *CEN1* and *CEN2* were marked with yellow and purple stars on the PFGE gel.

We amplified the *CEN2* loci with the OP44 and OP45 primers from all studied strains: Y879, Y881, Y997, Y891, Y880, Y865, Y883, Y901 (Table 1; S2 Fig). These strains have different karyotypes (Fig 4). Further analysis of our *D*. *bruxellensis* strains showed that MITE transposon (*CEN2-2*) is often located next to *CEN2*: in fact, we found *CEN2-2* in the strains Y881, Y997, Y865, Y883, Y901, and Y900 (S2 Fig). The cloned *CEN2* from Y879 strain, which lacks this transposon in this locus (plasmid P1210), displayed similar centromere properties as the *CEN2* from Y881 stain (Figs 2 and 3, S1 Fig).

The *CEN1* and *CEN2* loci hybridized to different chromosomes of *D*. *bruxellensis* (Fig 4). In some strains, the *CEN* loci hybridized to a few chromosomes. For example, in the Y891 strain, *CEN1* hybridized to two chromosomes, but in other strains it hybridized to one chromosome (Fig 4). The *CEN2* locus occurred in more chromosomes: It hybridized to three chromosomes in the strain Y883, to two chromosomes in the strains Y881, Y891, Y900, and Y901, and to one chromosome in the strains Y879, Y880, and Y865 (Fig 4). *When the CEN2 locus occurred in two or three chromosomes*, *the CEN2 locus always was close to a MITE transposon* (S2 Fig).

#### Conserved elements characteristic to centromeres

**Fig 5:**
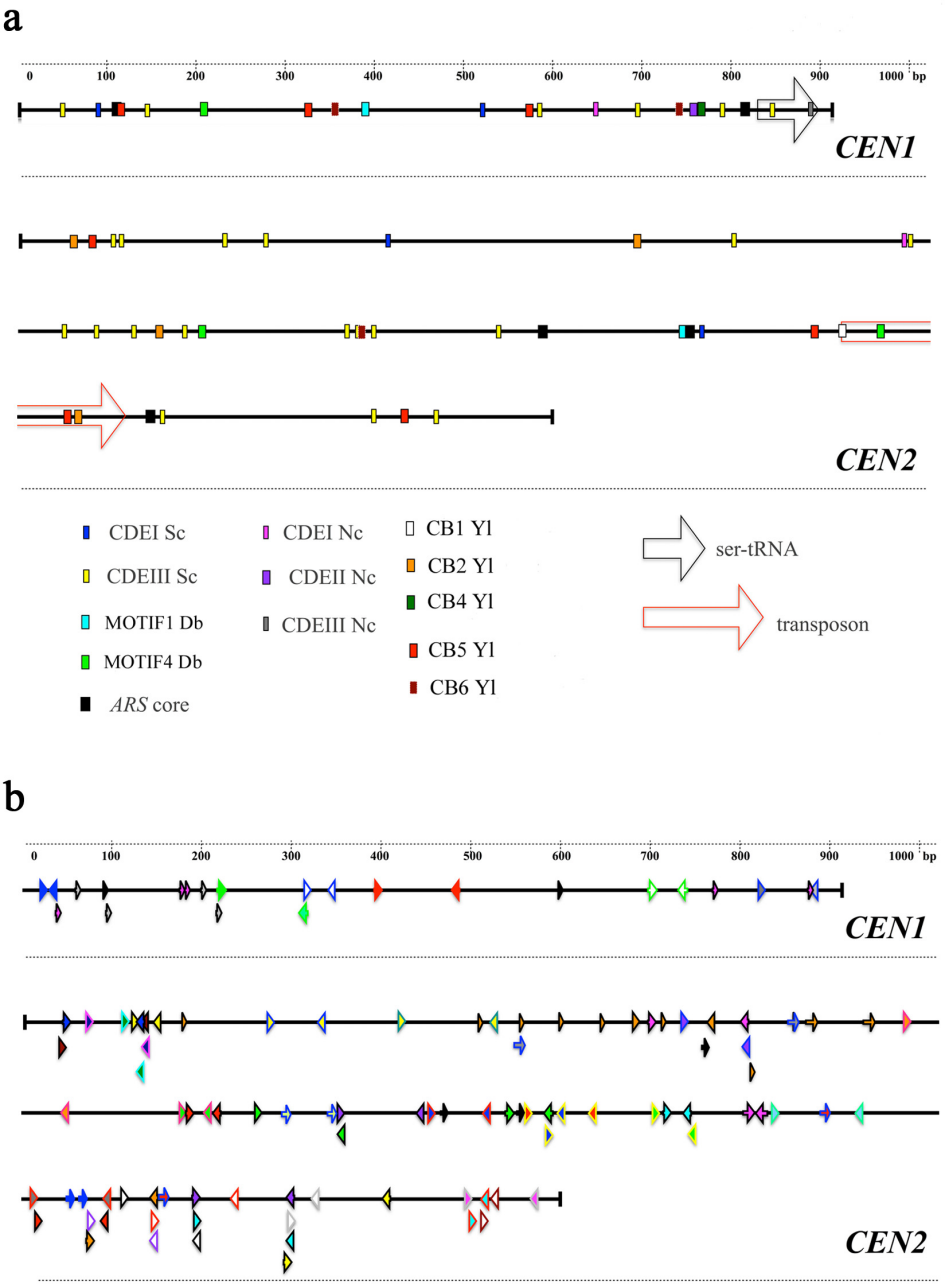
Direct and inverted repeats and motif sequences in *CEN1* and *CEN2*. (a) CDE-, *ARS-*, CB-like elements, *D*. *bruxellensis* specific motifs, ser-tRNA gene, and transposon-like sequences are shown. Sc–*S*. *cerevisiae*; Db–*D*. *bruxellensis*; Nc–*N*. *castellii*; Yl–*Y*. *lipolytica* (b) Direct and inverted repeats are shown on the *CEN1* and *CEN2* sequence. Repeats of the same type are shown with the same colour pattern.

*CEN1* and *CEN2* were searched for the presence of DNA elements typical for the yeast “conventional point” centromeres (found for example in *S*. *cerevisiae* and *Kluyveromyces lactis*): CDEI-like (TCACATG or TCACGTG) and CDEIII-like (TCCGAA) elements. Allowing for 1 base-pair mismatch in our search, several CDEI- and CDEIII-like elements were found in *D*. *bruxellensis CEN* loci (Fig 5a; S3 Table). In *S*. *cerevisiae* centromeres, CDEI is separated from CDEIII by the short AT-rich (>86%) CDEII region of 83–86 bp; no similar configuration was found in the *D*. *bruxellensis* sequences. The search for the CDE-elements of “unconventional” centromeres [18] revealed that the *D*. *bruxellensis CEN1* locus contains CDEI (GGGTAA, position 646–651 bp), CDEII (ACGGTTAT, position 755–762 bp) and CDEIII (TCCT, position 887–890 bp), as shown in S3 Table. Although these elements were separated by high-AT content DNA (73% of AT between CDEI and CDEII and 63% of AT between CDEII and CDEIII) in *D*. *bruxellensis*, their distances were greater in *D*. *bruxellensis* (103 and 124 bp) than in *N*. *castellii* (17–18 and 34 bp) [18].

Our *CEN*s were similar to centromeric loci in other yeast species. *D*. *bruxellensis CEN1* and *CEN2* carry conserved blocks (CBs), which are found in *Yarrowia lipolytica* centromeres [42]. Most *Y*. *lipolytica* CBs (except for CB3) were found in *CEN1* and *CEN2* loci with 1–2 base pair mismatches (Fig 5a; S3 Table).

To detect other elements common to both of the *D*. *bruxellensis CEN* loci, we searched for short motifs (at least 8 bp long) and found two new motifs, MOTIF1 (ACTTGGAG) and MOTIF4 (TACTTGAA) (Fig 5a; S3 Table). One of the MOTIF4 sequences was present within the transposon part *CEN2-2*. When we analysed a few randomly selected genomic loci from *D*. *bruxellensis* CBS 2499 database, we found hits of CDEI- and CDEII-elements of *S*. *cerevisiae* within the distance of 1 kb; however, no hits were found in 1 kb neighbourhoods of MOTIF4, which occurs in both *CEN1* and *CEN2* loci (data not shown).

#### *ARS*-like sequences

Sequence analysis of *CEN1* and *CEN2* also revealed the presence of several regions, which agreed with the consensus sequence of autonomous replicating sequences (*ARS*), *ARS* core and *ARS* box (Fig 5a; S3 Table), functional in *S*. *cerevisiae* [43–45], *C*. *albicans* [43], *Pichia quilliermondii* [46] and *Y*. *lipolytica* [47]. On the other hand, as discussed above, the *S*. *cerevisiae CEN/ARS* locus does not apparently operate in *D*. *bruxellensis*. Moreover, the circular *CEN1* and *CEN2* plasmids do not promote the transformation of *S*. *cerevisiae* (S4 Table).

#### Repetitive elements

When we analysed the *CEN* loci with a database of known repetitive DNA elements (GIRI repbase), *CEN1* and *CEN2* had short sequences (40–90 bp long) with 76%-89% homology to several transposable elements (DNA transposons Helitron, Harbinger, Polinton, Transib, LTR retrotransposon Gypsy, non-LTR retrotransposons) from different organisms (*Xenopus tropicalis*, *Caenorhabditis briggsae*, *Anolis carolinensis*, *Puccinia striiformis*, *Hydra magnipapillata*, *Tupaia belangeri*), as shown in S3 Table. As mentioned above, we also found a new transposon in the *D*. *bruxellensis CEN2* locus (*CEN2-2*, 1925–2120 bp; Fig 3e), which is similar to MITEs, which have been found in only plant and animal genomes [40, 41] but not in yeast.

Both *CEN* loci were found to carry several short inverted repeats (8–13 bp) and direct repeats (5–13 bp), as shown in Fig 5b and S5 Table. The complemented pairing of the repeats could result in numerous DNA loops and recombination spots in both *CEN1* and *CEN2*. The sequences of direct repeats did not overlap between *CEN1* and *CEN2*, except for the 13 bp repeat in *CEN2* CTGAAAGAGAAAT, which is present in *CEN1* but with 3 base-pair mismatches: AATTTTCTACCAA (direct repeat, position 35–47 bp), TGGGAAGAGGACT (reverse complement, position 446–458 bp), with consensus sequence _R_TGG_R_AGA_YR/Y_A_A/T_T.

### Induction of chromosomal breaks and association with centromeric histone H3

**Fig 6:**
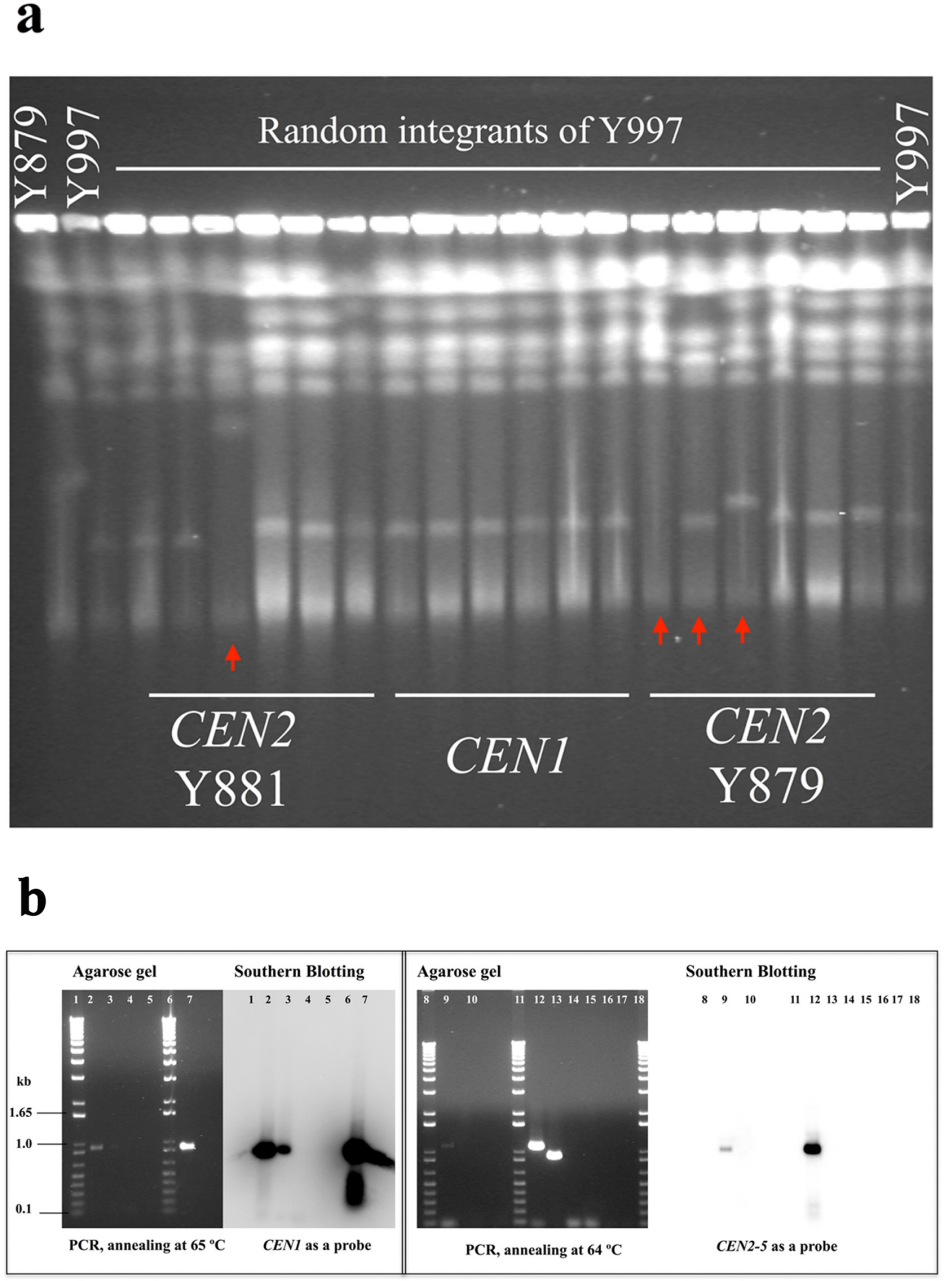
Chromosomal-break assay and association with the centromere histone H3. (a) Karyotype analysis of *D*. *bruxellensis* transformants carrying *CEN1* and *CEN2* plasmids integrated into the genome by PFGE. Y879 and Y997 strains were used as controls. Transformants with changed karyotypes are arrowed. (b) Co-immunoprecipitation with Cse4 antibodies. ChIP samples were used as a template for PCR performed at two annealing temperatures (64 and 65°C) using specific primers for *CEN1* (OP98 and OP99) and the sub-cloned fragment of *CEN2* (*CEN2-5*, primers SW9 and SW10). Samples: 1) 1 kb ladder; 2) ChIP Ca antibodies with Y879 chromatin (primers OP98 and OP99); 3) ChIP Ca antibodies with Y879 chromatin (primers OP98 and OP99); 4) ChIP Mouse Ig, negative control antibody with Y879 chromatin (primers OP98 and OP99); 5) no antibody, negative control with Y879 chromatin (primers OP98 and OP99); 6) 1 kb ladder; 7) input of ChIP assay, Y879 chromatin (primers OP98 and OP99); 8) 1 kb ladder; 9) ChIP Ca antibodies with Y879 chromatin (primers SW9 and SW10); 10) ChIP Ca antibodies with Y879 chromatin (primers OP98 and OP99); 11) 1 kb ladder; 12) input of ChIP assay, Y879 chromatin (primers SW9 and SW9); 13) input of ChIP assay, Y879 chromatin (primers OP98 and OP99); 14) ChIP Mouse Ig, negative control antibody with Y879 chromatin (primers SW9 and SW10); 15) no antibody, negative control with Y879 chromatin (primers SW9 and SW10); 16) ChIP Mouse Ig, negative control antibody with Y879 chromatin (primers OP98 and OP99); 17) no antibody, negative control with Y879 chromatin (primers OP98 and OP99); 18) 1kb ladder.

When a second centromere is integrated into the chromosome, the fate of this dicentric chromosome depends on the organism and its stability varies [48]. In budding yeasts and in the fruit fly, dicentric centromeres break the chromosome or rearrange themselves [48–53]. Under some conditions, if the same spindle pole is used for both centromeres, then this chromosome is successfully propagated to the progeny [48, 54, 55]. The second centromere on the chromosome can be also inactivated [48, 55–58]. To investigate whether cloned loci *CEN1* and *CEN2* have these centromeric properties, we integrated plasmids carrying these loci into the Y997 genome and analysed the karyotype of obtained integrants by PFGE. As transformation by homologous recombination is not yet developed in *D*. *bruxellensis*, we generated random integrants from linearized *CEN* plasmids through non-homologous integration into the genome. The *CEN1* integrants did not display differences in karyotype with recipient strain Y997 (Fig 6a), while the *CEN2* plasmids (P950 and P1210, with or without transposon, respectively) did induce chromosomal breaks in some of the transformants resulting in different chromosome patterns (Fig 6a). The *CEN1* integration did not result in any chromosome breaks visible by PFGE. As *CEN1* did not induce chromosomal breaks but had centromeric properties when cloned on the plasmid, we induced no further sub-cloning. As *CEN2* induced chromosomal breaks, we performed its further sub-cloning (Supporting Information). Among the *CEN2* deletion plasmids, only the sub-cloned fragment *CEN2-5* supported plasmid autonomous replication (Fig 3d); however this fragment failed to induce chromosomal breaks (data not shown). Although both *CEN2-5* and *CEN1* have centromeric properties when cloned on the circular plasmid, they have smaller size and have fewer repeats on its sequence than does *CEN2*. The reduced size with fewer repeats on their sequence could result in reduced centromeric function (for example, an inability to cause chromosomal breaks when integrated on the chromosome). For the *CEN1*, the bigger fragment carrying neighbour genomic loci needs further study.

To investigate whether the isolated *CEN1* and *CEN2* loci associate with the centromeric histone H3 (CenH3), we performed chromatin co-immunoprecipitation assay. The gene coding for CenH3 is not known in *D*. *bruxellensis*. The search using the *S*. *cerevisiae* and *C*. *albicans CSE4* gene sequences (encoding CenH3) revealed few hits. However, in the case of *C*. *albicans*, the sequence identity was higher than that with *S*. *cerevisiae*. For this reason we decided to use antibodies raised against *C*. *albicans* Cse4 (Materials and Methods section). The chromatin was prepared from the Y879 strain, sheared by sonication and incubated with antibodies raised against *C*. *albicans* Cse4 (Materials and Methods section). The chromatin-bound antibodies were purified from the reaction by coupling them to magnetic beads. This was followed by PCR analysis using specific primers for *CEN1* (OP98 and OP99) and for the *CEN2-5* part of *CEN2* (SW9 and SW10) as shown in Fig 6b and S1 Table. The antibodies pulled out a fragment of the expected size for *CEN1* (0.916 kb). The fragment’s identity was confirmed by Southern blotting (Fig 6b) and by sequencing. Similarly, the antibodies pulled out the expected *CEN2-5* fragment (1.1 kb), the identity of which was confirmed by Southern blotting (Fig 6b) and sequencing.

### Determination of plasmid copy and development of a heterologous expression system

To estimate the copy number and expression properties of our *CEN* plasmids, we developed a reporter gene system using the *Kluyveromyces lactis LAC4* gene (S1 Materials and Methods). The Y997 did not display any native β-galactosidase activity (S3 Fig) and was selected for the expression of heterologous gene cloned under strong promoter (S4 Fig). The RT-PCR was performed using genomic DNA of the *CEN* replicative transformants as a template and control strain Y1377 carrying one copy of the reporter cassette with the *LAC4* and *URA3* genes (S5 Fig). As shown in S6 Fig and S6 Table the *CEN* transformants were found to carry 1–1.5 plasmid copy per cell. In spite of the same copy number the expression of a heterologous gene, *LAC4*, was 24.5 times higher from the replicative *D*. *bruxellensis CEN2* plasmids than from the reporter plasmid integrated into the genome (S7 Fig), which could result from the silencing of the gene integrated into the genome. The presence of a transposon in the replicative *CEN2* plasmid improved the expression of a reporter gene slightly; the corresponding activity was 1.2-fold higher when expressed from P1211 (*CEN2* from Y881; S7 Fig).

### Transformants carrying replicative plasmids with *CEN1* and *CEN2* undergo phenotypic switch

**Fig 7:**
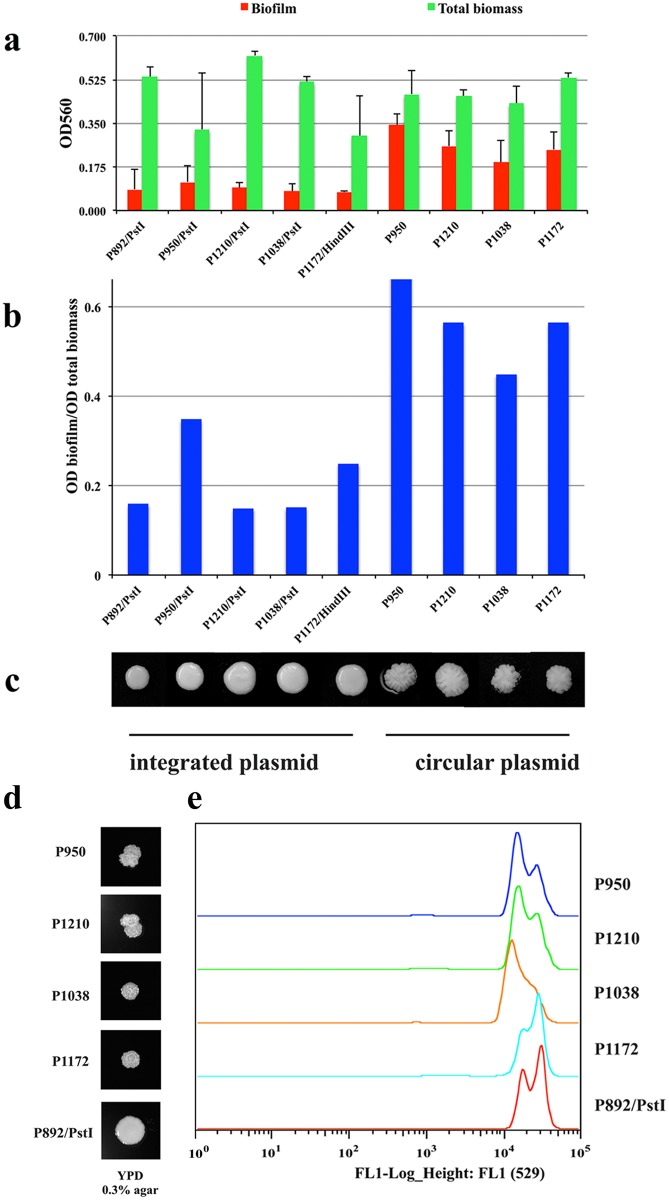
Biofilm, mat formation, and colony morphology of Y997 transformants carrying *CEN1* and *CEN2* plasmids. (a) Biofilm and total biomass of transformants grown in YNB with 0.2% glucose for 48 h. Transformants analysed: (1) integrative (P892/*Pst*I (control strain carrying empty vector); P950/*Pst*I (*CEN2* with transposon); P1210/*Pst*I (*CEN2* without transposon, from Y879 strain); P1038/*Pst*I (*CEN2-5* fragment with transposon); P1172/*Hind*III (plasmid carrying *CEN1*)); (2) replicative (P950 (*CEN2* with transposon); P1210 (*CEN2* without transposon, from Y879 strain); P1038 (*CEN2-5* fragment with transposon); P1172 (*CEN1*)). (b) The biofilm normalized by biomass. (c) The colony morphology of corresponding transformants on YNB medium with 2% glucose and 2% agar. (d) Mat formation by Y997 transformants on YPD with 0.3% agar grown 30 days at room temperature. (e) The DNA content of each transformant carrying circular plasmid estimated by flow cytometry and compared to that of the control strain P892/*Pst*I (P892 integrated into the genome). P950; P1210; P1038 and P1172.

The untransformed strain Y997 (*ura3* mutant) formed mainly smooth colonies. Only one fluffy colony of Y997 was found per 200 studied on the media with glucose, which could be due to prions, as no fluffy colonies were formed on guanidine hydrochloride (S8 Fig). The integrative transformants of Y997 formed colonies with smooth surfaces similar to the control strain, which carried the empty plasmid P892 with *URA3* gene (Fig 7). Strikingly, the transformants carrying the *CEN1* and *CEN2* replicative plasmids underwent phenotypic switch, as their colony surface was fluffy (Fig 7c). This “fluffy” phenotype was not possible to resolve by the treatment of guanidine hydrochloride (S9 Fig) and thus was not associated with any prions. The “fluffy” phenotype in transformants carrying circular, replicative plasmids with either *CEN1* or *CEN2* could be due to the change in the gene expression responsible for biofilm. Among the plasmid integrants, there was no clear difference in biofilm formation or in colony morphology (Fig 7). The full length of *CEN2* (P950 and P1210) was found earlier to contain putative MATE family drug/sodium antiporter. Although MATE family drug/sodium antiporters contribute to biofilm and fluffy colonies in *S*. *cerevisiae* [59], the integrants of *CEN2* (P950 and P1210) were not clearly different than the control strain and *CEN1* and other integrants, which do not carry MATE genes. In contrast, all replicative transformants carrying both *CEN1* and *CEN2* loci form clearly more biofilm (2.8–3.6 times higher) than do the integrants and the control strain with empty vector integrated into the genome (Fig 7a and 7b). In addition to having the “fluffy” phenotype and improved biofilm production, the replicative transformants formed more structurally complex mats when transferred into low agar medium (Fig 7d). Thus the biofilm and the “fluffy” phenotype are due to the circular (replicative) plasmid status in yeast transformants. The karyotype analysis by PFGE did not show any detectable changes in chromosome pattern in these transformants compared to the recipient strain (data not shown). The distinguishing feature of the transformants carrying circular plasmids *CEN1* and *CEN2* was found to be the shifts in ploidy, since their DNA content was altered (Fig 7e). The recipient strain Y997 is diploid [32] and the loss or gain of one of the chromosome copy is not easy to monitor by PFGE. On the other hand, as detected by flow cytometry, the transformants with the *CEN2* plasmid displayed decreased DNA content, while *CEN1* transformants had slightly increased DNA content (Fig 7e); both of these changes could be due to the aneuploidy. Yeast aneuploidy, which contributes to changes in colony morphology in *S*. *cerevisiae* [60], is a plausible cause of the observed phenotypic switch in replicative *CEN1* and *CEN2* transformants of *D*. *bruxellensis*.

## Discussion

The genome of the pre-WGD yeast *Dekkara bruxellensis* has high variability in its karyotypes and ploidy (among isolates) [27]. This study investigated the yeast’s genomic architecture to understand its high karyotype variability. As part of this investigation, we cloned and characterized essential chromosome regions.

We constructed a gene library, from which we isolated two *D*. *bruxellensis CEN* loci that associate with the centromeric histone H3 (CenH3). The loci *CEN1* and *CEN2* were shown to be AT-rich and (when transformed into *D*. *bruxellensis*) to have centromere properties. Only if a plasmid carries a functional centromere, then it behaves like a chromosome, which by definition is stably maintained during mitosis and meiosis [8, 9]. The centromeres *CEN1* and *CEN2* provided plasmid mitotic stability with plasmid loss per generation between 0.4%–2.0% when no selection pressure was applied. This mitotic stability is similar to the stability of the plasmids carrying centromeres of the yeasts *S*. *cerevisiae* and *Y*. *lipolytica*, which display 1%–3% and 1%–4% loss per generation respectively [12, 47]. Unlike centromeres, *ARS*-carrying plasmids are unstable without selection pressure; without such pressure, they display 5%-50% plasmid loss [61, 62]. This evidence strongly suggests that *CEN1* and *CEN2* are actual centromeres, because no other structure capable of promoting stable plasmid maintenance and association with centromeric histone. Of course, kinetochore formation is another defining property of the centromere, and it remains to be studied for *CEN1* and *CEN2*.

Besides their defining properties, centromeres are distinguished from *ARS*s by secondary phenomena. In *S*. *cerevisiae*, centromere-based plasmids are present in one or two copies per genome [8, 12, 17, 63]. In *Y*. *lipolytica*, the copy-number is around 3 per cell [42, 47]. Unlike centromeres, *ARS*-containing plasmids often have copy numbers around 50–100 [64, 65]. When a plasmid has both a centromere and an *ARS*, the plasmid copy-number is 1–2 copies per cell. In our study, the *CEN* plasmids had (on average) 1.5 copies per cell, further strengthening the credibility of the finding that *CEN1* and *CEN2* are centromeres.

The *D*. *bruxellensis* loci *CEN1* and *CEN2* differ in structure from the centromeres of other species. They have features of both “point” and “regional” centromeres.

For “point” centromeres, their functions are determined by the sequence of each centromere. Conserved short motifs were found within both *CEN1* and *CEN2*. For example, in *S*. *cerevisiae* a short AT-rich CDEII region separates CDEI and CDEIII elements [14]. However, no homologue to this CDEII was found in *D*. *bruxellensis*: on the other hand, CDEI was present in 2 copies and also CDEIII was present in 6 and 18 copies in *CEN1* and *CEN2* respectively. Surprisingly, the three CDE motifs claimed to be characteristic of “unconventional” centromeres [18] were found, each in 1 copy, in *CEN1*. However the AT-dense regions between CDEI, CDEII and CDEIII were longer in *CEN1* and *CEN2* than those that had been reported for *N*. *castellii* [18]. There are also novel 8 bp long motifs present on both *CEN* loci in 1–2 copies, which could represent conserved elements specific to the centromeres of *D*. *bruxellensis* centromere. We found the ser-tRNA gene near the *D*. *bruxellensis* centromere, which is not common for “point” centromeres and has been reported for only *N*. *castellii CEN7* [18].

The *CEN* loci of *D*. *bruxellensis* have features of the “regional” centromeres reported for *Y*. *lipolytica* and *C*. *albicans* [6, 47, 66]. For example, *CEN1* and *CEN2* carry *ARS* elements. In *Y*. *lipolytica*, *ARSs* and centromeres together form an active *ARS* while the centromeres supply the partition system required for *ARS* function [42]. *CEN1* and *CEN2* were enriched with repetitive DNA like the centromeres of *Y*. *lipolytica* and *C*. *albicans* [7, 42]. Several short inverted (8–13 bp) and direct (5–13 bp) repeats were present in both *CEN* loci. Direct and inverted repeats play a role in the function of centromeres [67]. For example, the recombination at the repeated centromere loci of the fission yeast *S*. *pombe* results in covalently closed DNA loops [67], which could provide boundaries for the centromeric histone H3 [67]. In other yeasts, recombination at the repeats leads to the double-stranded breaks occuring in isochromosomes and in neo-centromere formation [38, 67, 68].

The transposon near *CEN2* resembles the miniature inverted—repeat transposable elements (MITE) found in higher eukaryotes [69, 70], which have not been reported for yeasts. As with other transposons, *CEN2*’s MITE-like transposon is likely to promote genome rearrangements, particularly because of this association: *In all the D*. *bruxellensis strains that carried the transposon near CEN2*, *the CEN2 was duplicated*.

Our transformants carrying circular plasmids with *CEN1* or *CEN2* switched their colony morphology. Such phenotypic switching is associated with aneuploidy in *S*. *cerevisiae* [60] and, we report, for *D*. *bruxellensis*: Our transformants also changed ploidy from diploid to aneuploid. The introduction of the centromeric plasmid changes the ploidy and so imbalances the chromosome copy. All of these rearrangements change genes expression, perhaps explaining the observed changes in biofilm formation and colony morphology [71, 72].

The molecular mechanisms of the centromeres of *D*. *bruxellensis*, particularly of kinetochore formation, need further study. *However*, *our study establishes the proposition that centromeric regions (particularly CEN1 and CEN2) change the karyotype and the ploidy of D*. *bruxellensis*.

The presence of the transposon-like element near *CEN2* and the presence of other repeats at both *CEN1* and *CEN2* suggest an alternative mechanism of increase in chromosome number and genome plasticity. Repetitive elements (*i*.*e*., transposons) could serve as “hot spots” for recombination that could lead to aneuploidy. Non-replicative transposition of transposons leave a break in the donor site, which once healed could lead to the formation of new chromosomes.

## Dedication

Dedicated to the blessed memory of Professor Jure Piškur (1960–2014).

## Supporting Information

S1 Fig Analysis of *CEN1* and *CEN2* plasmids isolated from yeast transfromants through shuttling to *E*. *coli*. Plasmids native forms and their restriction pattern obtained with *Eco*RI were analyzed. a) P950 (*CEN2* from Y881 strain carrying transposon); b) p1210 (*CEN2* from Y879 strain without transposon); c) P1038 (*CEN2* deletion plasmid *CEN2-5*); d) P1172 (*CEN1*).
(TIF)

S2 Fig Amplification of *CEN1*, *CEN2* and *CEN2* parts from the genome of different *D*. *bruxellensis* strains by PCR. a) *CEN1* was amplified with primers OP98 and OP99; b) *CEN2* (with and without transposon) was amplified using primers OP44 and OP45; c) *CEN2-5* was amplified with primers SW9 and SW10; d) *CEN2-1* with *CEN2-2* (transposon part) amplified with primers OP91 and OP94. Total DNA of the corresponding strains was used as a template for PCR: 1 –Y879, 2 –Y881, 3 –Y891, 4 –Y880, 5 –Y865, 6 –Y883, 7 –Y900, 8 –Y901, 9 –Y997, 10 –negative control.
(TIF)

S3 Fig The overlay for the qualitative assay of β-galactosidase (with X-Gal) on *D*. *bruxellensis* strains Y879 and Y997 grown on YP+lactose or YP+glucose. (TIF)

S4 Fig The linear presentation of plasmids P935, P1160 and P1211. The *URA3* gene of *D*. *bruxellensis* is shown as a grey box; *K*. *lactis LAC4* gene—red box; promoter *YPR100W* (*MRPL51*, Mitochondrial ribosomal protein of the large subunit)—yellow box; *CEN2*—black boxes (transposon *CEN2-2* is shown as blue box); pUC57 part—thin line. Restriction sites: RI, *Eco*RI; Sc, *Sac*I; K, *Kpn*I; Xb, *Xba*I; B, *Bam*HI; Sl, *Sal*I; P, *Pst*I; Sp, *Sph*I; H, *Hind*III.
(TIF)

S5 Fig Southern hybridization that illustrates the copy number of the plasmid P935 integrated into the genome of *ura3* mutant Y997. a) The fragment carrying *D*. *bruxellensis URA3* gene is shown as a grey box; the *K*. *lactis LAC4* gene—red arrow; *D*. *bruxellensis* promoter *YPR100W* (*MRPL51*, Mitochondrial ribosomal protein of the large subunit)—blue box; the genomic DNA of Y997 strain—wavy line. Prior the transformation procedure the plasmid was digested with *Sac*I (Sc). The genomic DNA of the transformants was digested with *Pst*I (P) and hybridized with [γ-^32^P] dCTP-labeled *LAC4* gene. b) 1—P935; 2 - 1kb DNA ladder (NEB); 3—genomic DNA of Y997 digested with *Pst*I; 4—genomic DNA of Y1377 digested with *Pst*I; 5—genomic DNA of Y1378 digested with *Pst*I.
(TIF)

S6 Fig Relative *URA3* gene copy number in *D*. *bruxellensis* transformants carrying circular *CEN1* and *CEN2* plasmids. RT-PCR data of *URA3* amplification were normalized within each strain by the data of *YML085C* encoding α-tubulin. Strains Y991 (one *URA3* gene copy) and Y1377 (two *URA3* gene copies) was used to calculate relative gene copy number (S6 Table). Transformants: P950 (*CEN2* of Y881); P1210 (*CEN2* of Y879 strain); P1038 (*CEN2-5* fragment of *CEN2*); P1172 (*CEN1*).
(TIF)

S7 Fig Quantitative β-galactosidase assay in D. bruxellensis transfromants. Y1377—transformant carrying one copy of P935 (*K*. *lactis LAC4* gene) integrated were used as control; transformants with replicative *CEN2* plasmids P1211 (carrying *CEN2* from Y881 strain) and P1160 (carrying *CEN2* from Y879) were used in the assay; ura+ transformant of recipient strain Y997 was used as negative control. The corresponding strains were grown in the liquid YNB with glucose as a carbon source.
(TIF)

S8 Fig Colony morphology of Y997 on the minimal media with and without guanidine hydrochloride. The fluffy colony is arrowed.
(TIF)

S9 Fig Colony morphology of transformants of Y997 with replicative plasmids with *CEN1* and *CEN2* on the minimal media with and without guanidine hydrochloride. (TIF)

S1 Materials and Methods Plasmids and molecular-biology techniques. Development of *LAC4* reporter system and β-galactosidase assay. Deletion studies of *CEN2*.
(DOCX)

S1 Table Oligonucleotides used in this study. (DOCX)

S2 Table The frequency of appearance of *CEN* loci in the *D*. *bruxellensis* CBS 2499 (Y879) genome (http://genome.jgi.doe.gov/Dekbr2/Dekbr2.home.html). (DOCX)

S3 Table Motifs found in *D*. *bruxellensis CEN1* and *CEN2*. (DOCX)

S4 Table Transformation efficiency of *S*. *cerevisiae* Y601 with plasmids carrying *D*. *bruxellensis CEN1* and *CEN2*. (DOCX)

S5 Table Inverted and direct repeats found in *CEN1* and *CEN2*. (DOCX)

S6 Table Estimation of relative *URA3* gene copy number in the *D*. *bruxellensis* Y997 transformants by RT-PCR. (DOCX)
