# Supplementary figures and images for: Novel Centromeric Loci of the Wine and Beer Yeast Dekkera bruxellensis CEN1 and CEN2
Source: PLoS One. 2016 Aug 25;11(8):e0161741. doi: 10.1371/journal.pone.0161741 (PMC4999066; doi:10.1371/journal.pone.0161741)

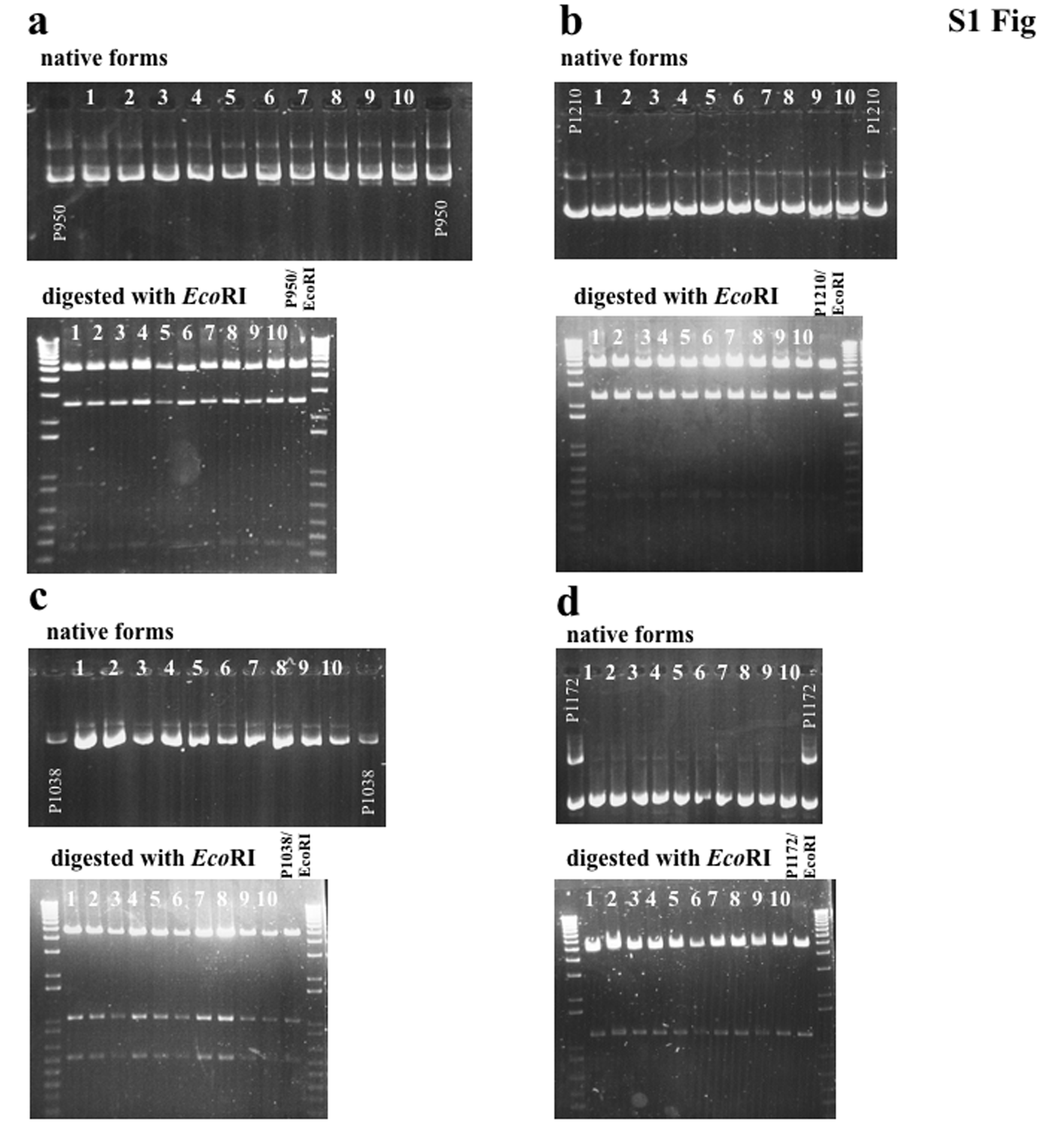

Supplement: S1 Fig — Plasmids native forms and their restriction pattern obtained with EcoRI were analyzed. a) P950 (CEN2 from Y881 strain carrying transposon); b) p1210 (CEN2 from Y879 strain without transposon); c) P1038 (CEN2 deletion plasmid CEN2-5); d) P1172 (CEN1). (TIF) [file pone.0161741.s001.tif]

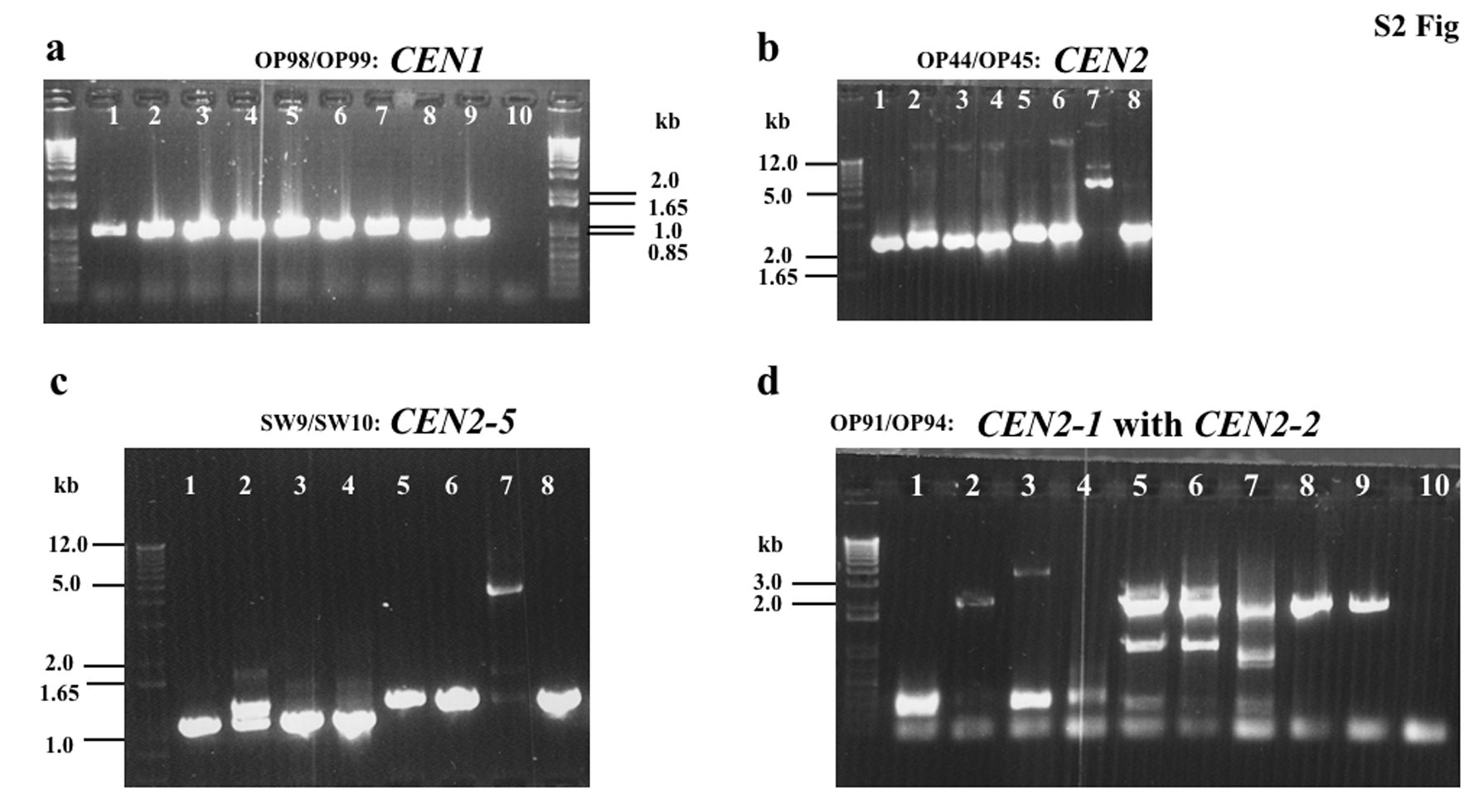

Supplement: S2 Fig — a) CEN1 was amplified with primers OP98 and OP99; b) CEN2 (with and without transposon) was amplified using primers OP44 and OP45; c) CEN2-5 was amplified with primers SW9 and SW10; d) CEN2-1 with CEN2-2 (transposon part) amplified with primers OP91 and OP94. Total DNA of the corresponding strains was used as a template for PCR: 1 –Y879, 2 –Y881, 3 –Y891, 4 –Y880, 5 –Y865, 6 –Y883, 7 –Y900, 8 –Y901, 9 –Y997, 10 –negative control. (TIF) [file pone.0161741.s002.tif]

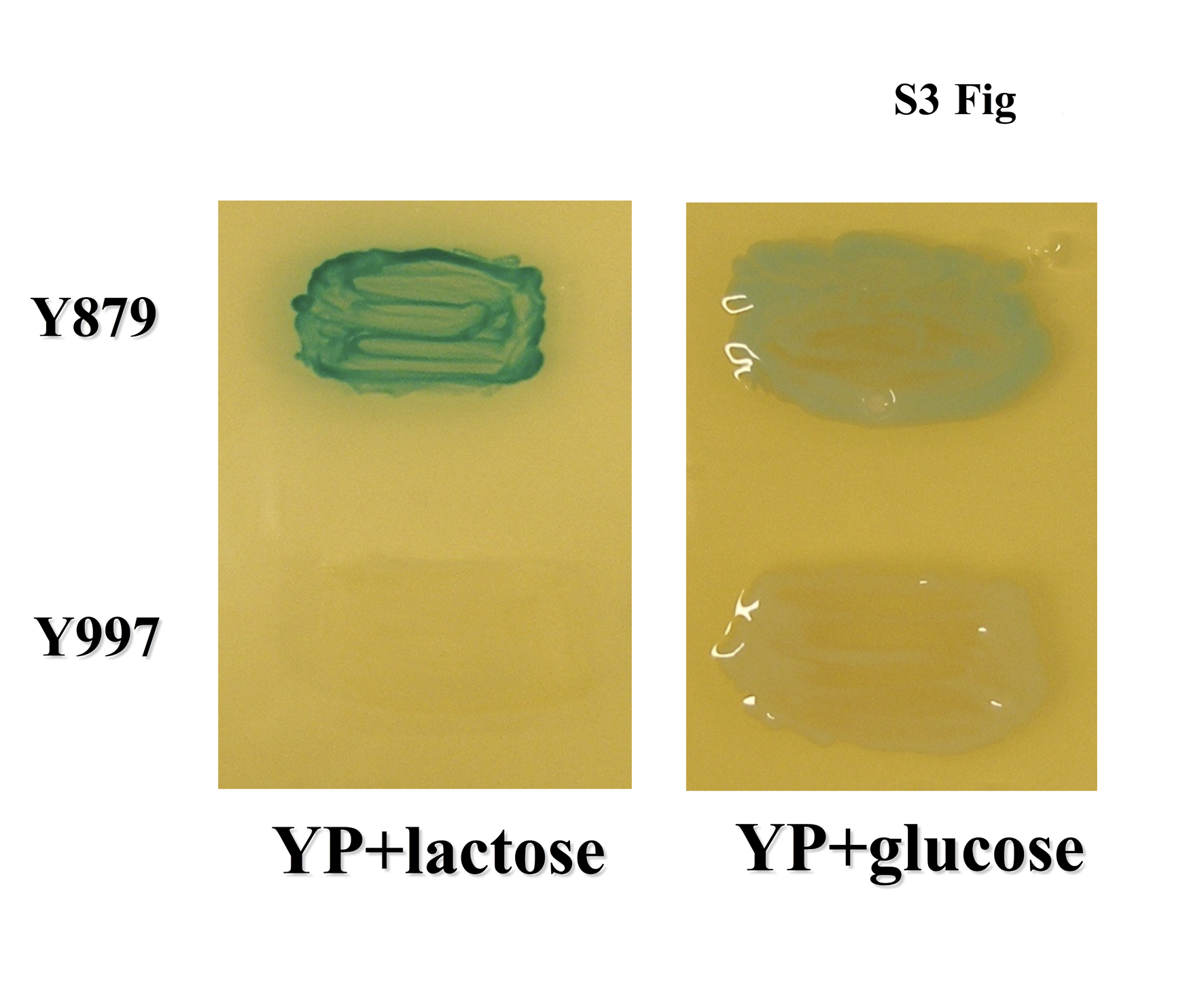

Supplement: S3 Fig — (TIF) [file pone.0161741.s003.tif]

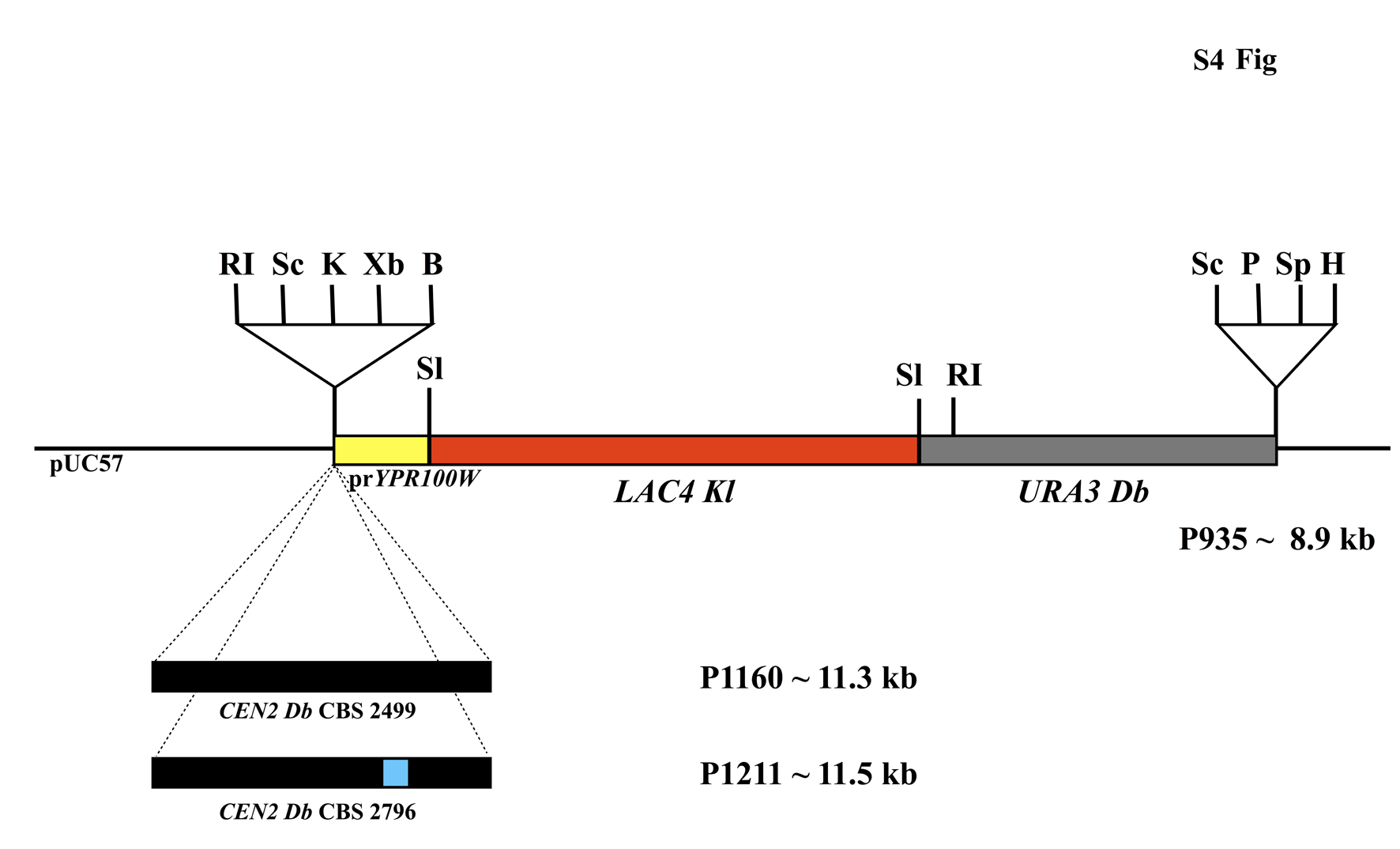

Supplement: S4 Fig — The URA3 gene of D. bruxellensis is shown as a grey box; K. lactis LAC4 gene—red box; promoter YPR100W (MRPL51, Mitochondrial ribosomal protein of the large subunit)—yellow box; CEN2—black boxes (transposon CEN2-2 is shown as blue box); pUC57 part—thin line. Restriction sites: RI, EcoRI; Sc, SacI; K, KpnI; Xb, XbaI; B, BamHI; Sl, SalI; P, PstI; Sp, SphI; H, HindIII. (TIF) [file pone.0161741.s004.tif]

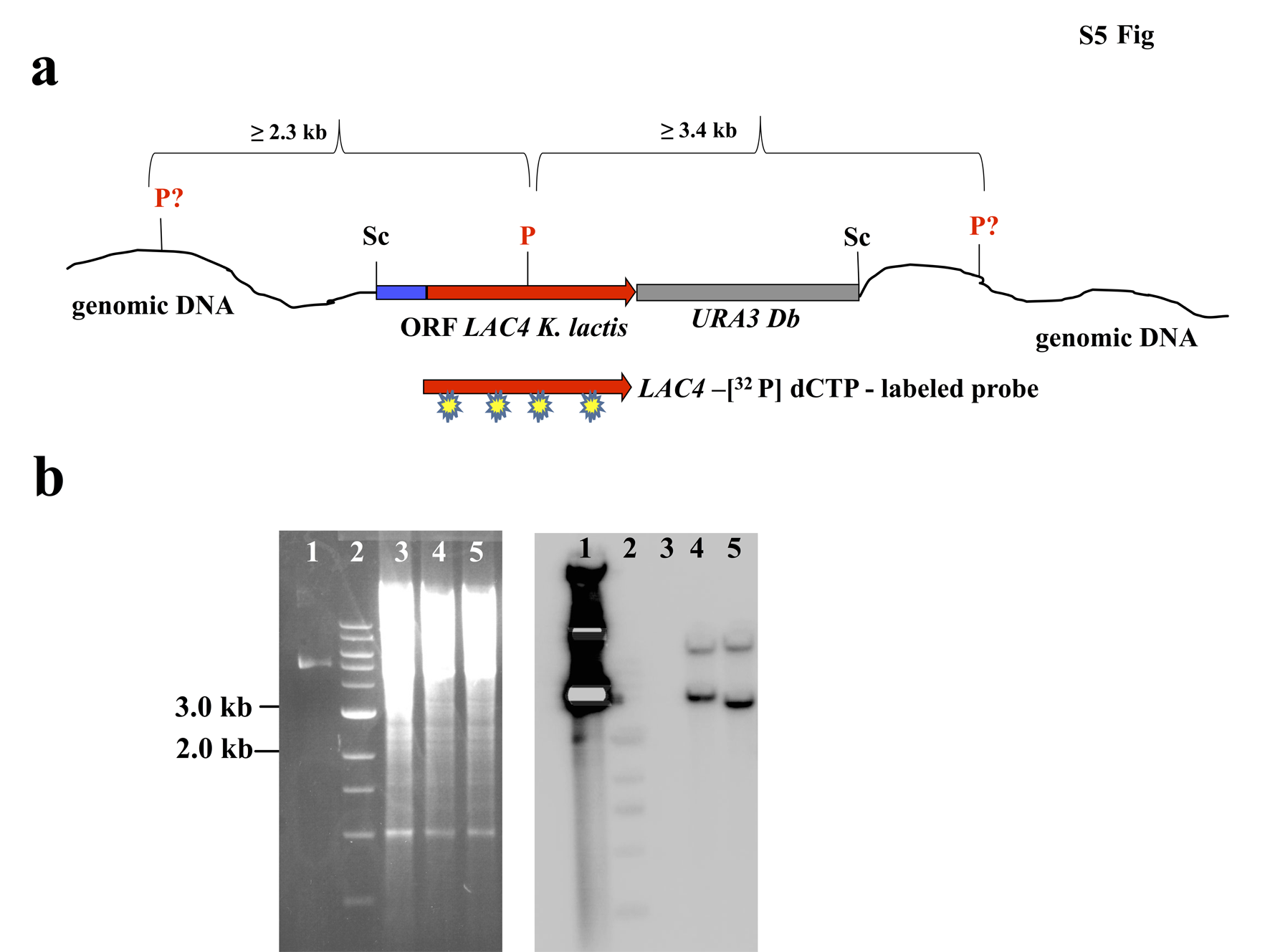

Supplement: S5 Fig — a) The fragment carrying D. bruxellensis URA3 gene is shown as a grey box; the K. lactis LAC4 gene—red arrow; D. bruxellensis promoter YPR100W (MRPL51, Mitochondrial ribosomal protein of the large subunit)—blue box; the genomic DNA of Y997 strain—wavy line. Prior the transformation procedure the plasmid was digested with SacI (Sc). The genomic DNA of the transformants was digested with PstI (P) and hybridized with [γ-32P] dCTP-labeled LAC4 gene. b) 1—P935; 2 - 1kb DNA ladder (NEB); 3—genomic DNA of Y997 digested with PstI; 4—genomic DNA of Y1377 digested with PstI; 5—genomic DNA of Y1378 digested with PstI. (TIF) [file pone.0161741.s005.tif]

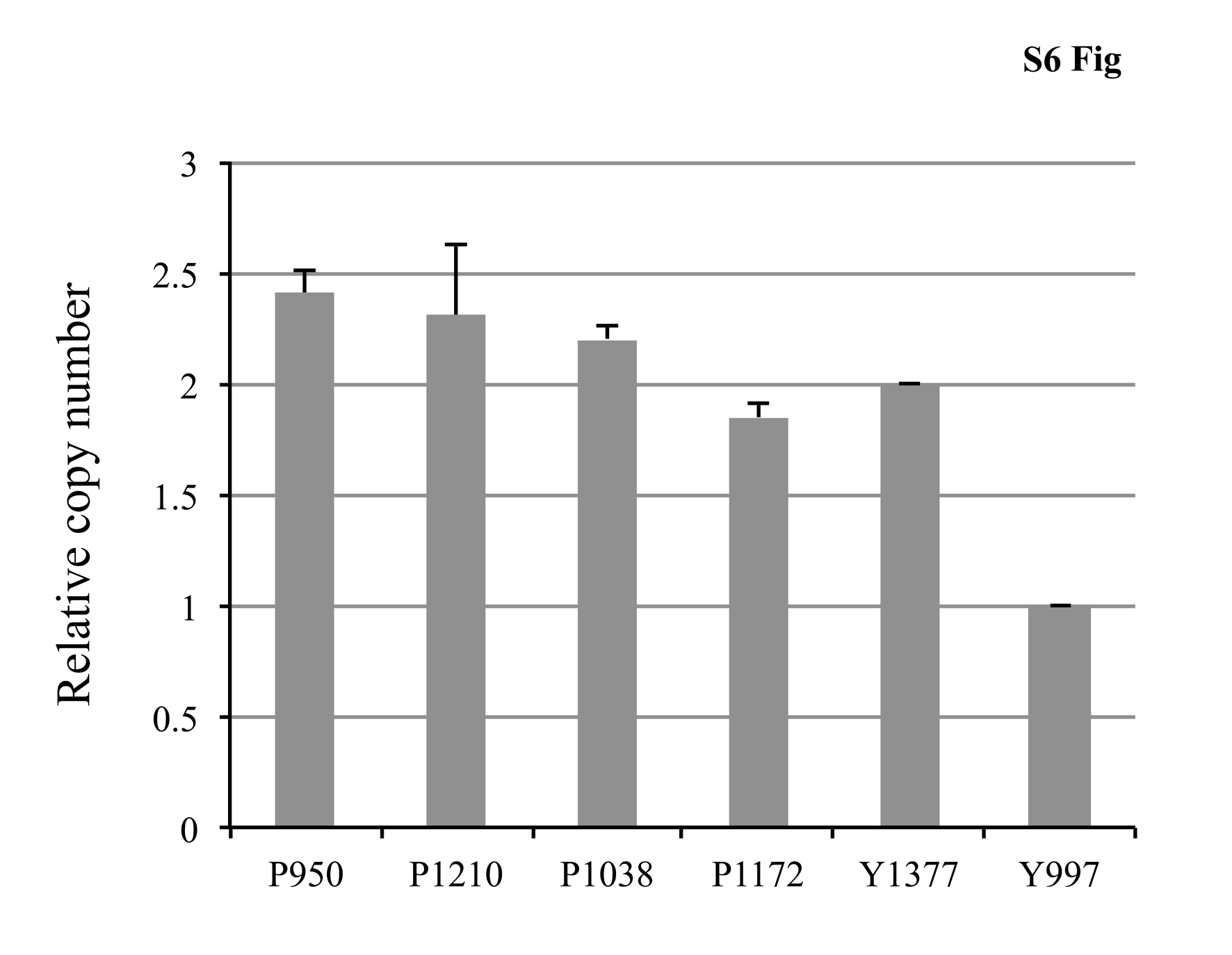

Supplement: S6 Fig — RT-PCR data of URA3 amplification were normalized within each strain by the data of YML085C encoding α-tubulin. Strains Y991 (one URA3 gene copy) and Y1377 (two URA3 gene copies) was used to calculate relative gene copy number (S6 Table). Transformants: P950 (CEN2 of Y881); P1210 (CEN2 of Y879 strain); P1038 (CEN2-5 fragment of CEN2); P1172 (CEN1). (TIF) [file pone.0161741.s006.tif]

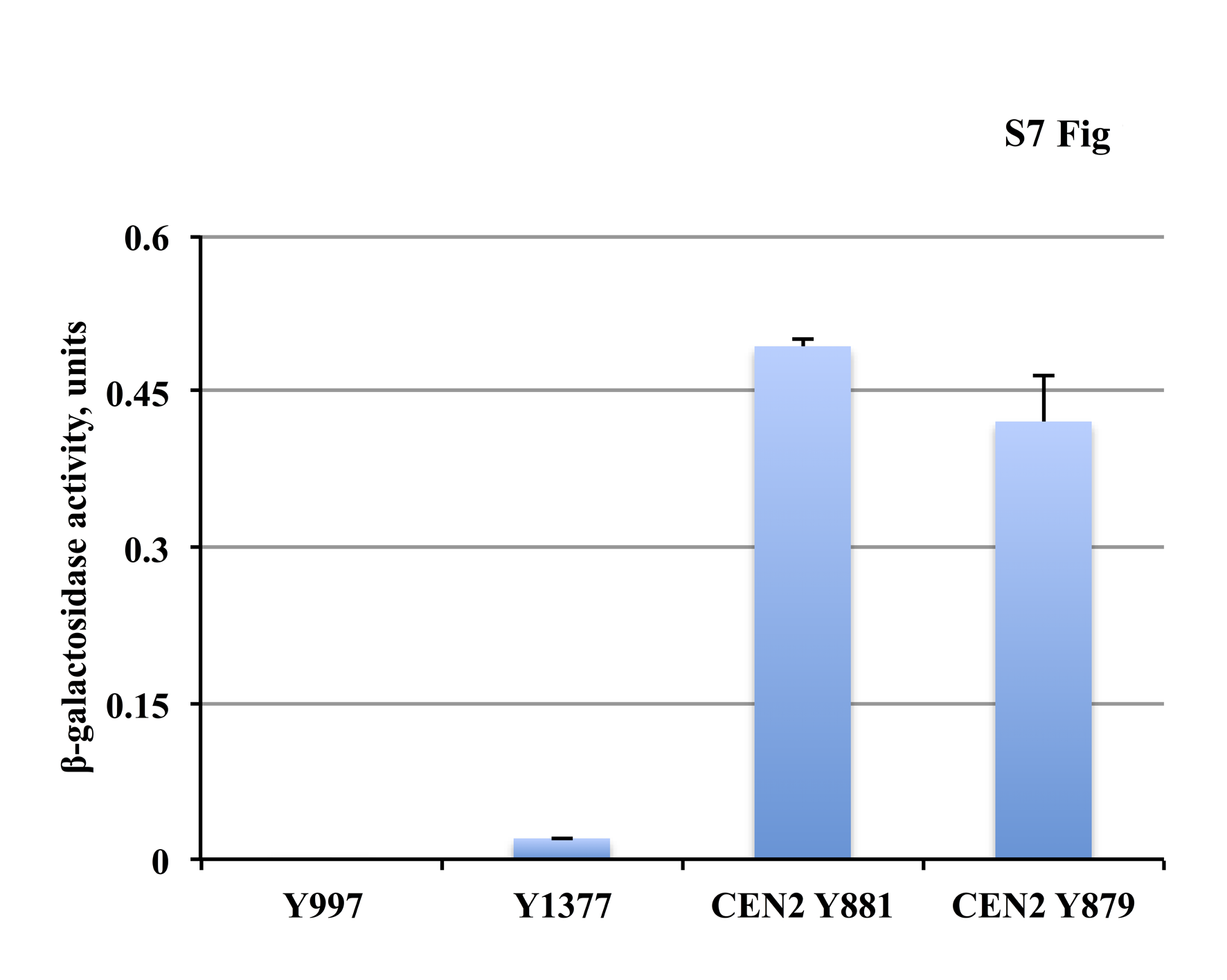

Supplement: S7 Fig — Y1377—transformant carrying one copy of P935 (K. lactis LAC4 gene) integrated were used as control; transformants with replicative CEN2 plasmids P1211 (carrying CEN2 from Y881 strain) and P1160 (carrying CEN2 from Y879) were used in the assay; ura+ transformant of recipient strain Y997 was used as negative control. The corresponding strains were grown in the liquid YNB with glucose as a carbon source. (TIF) [file pone.0161741.s007.tif]

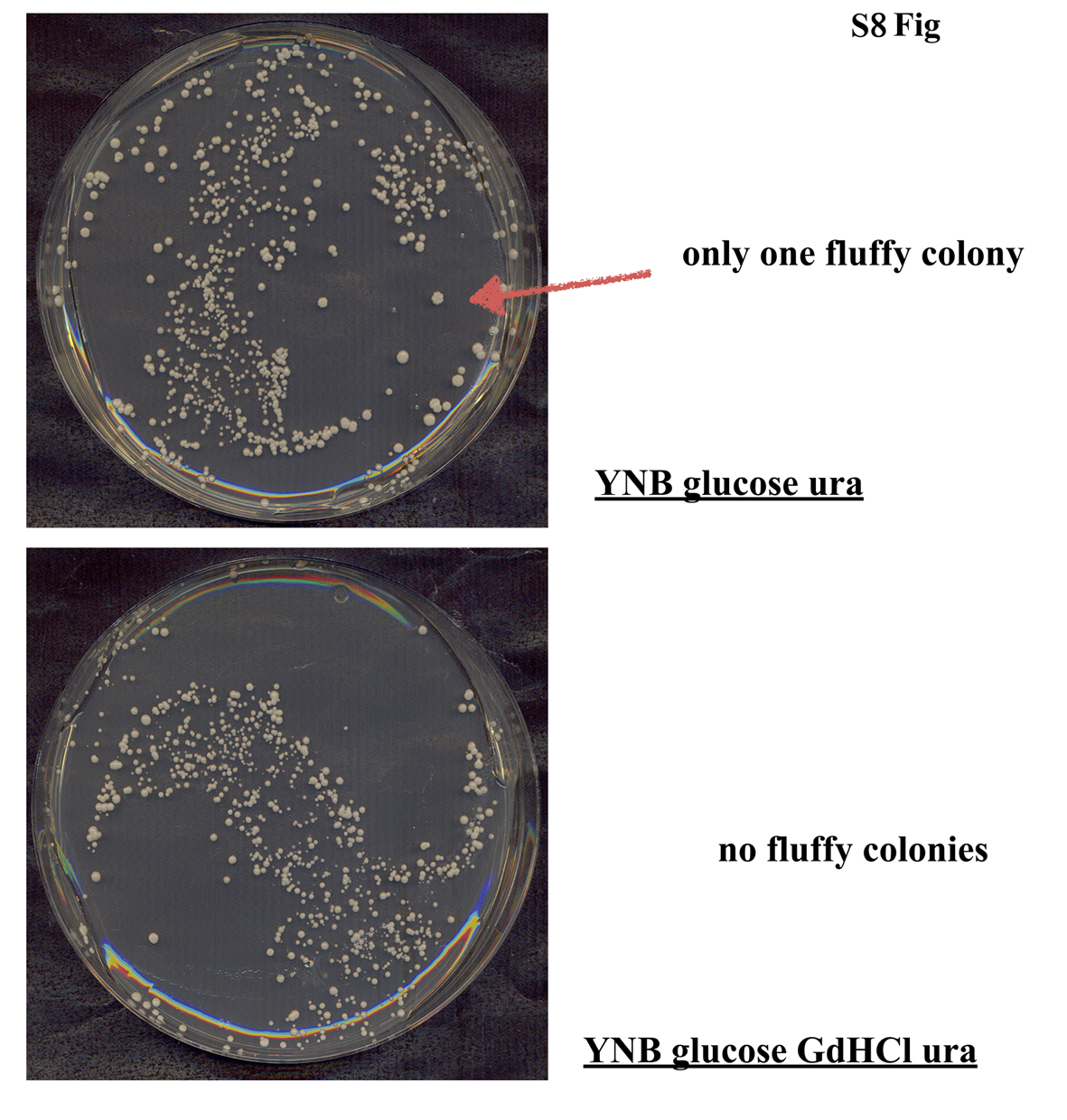

Supplement: S8 Fig — The fluffy colony is arrowed. (TIF) [file pone.0161741.s008.tif]

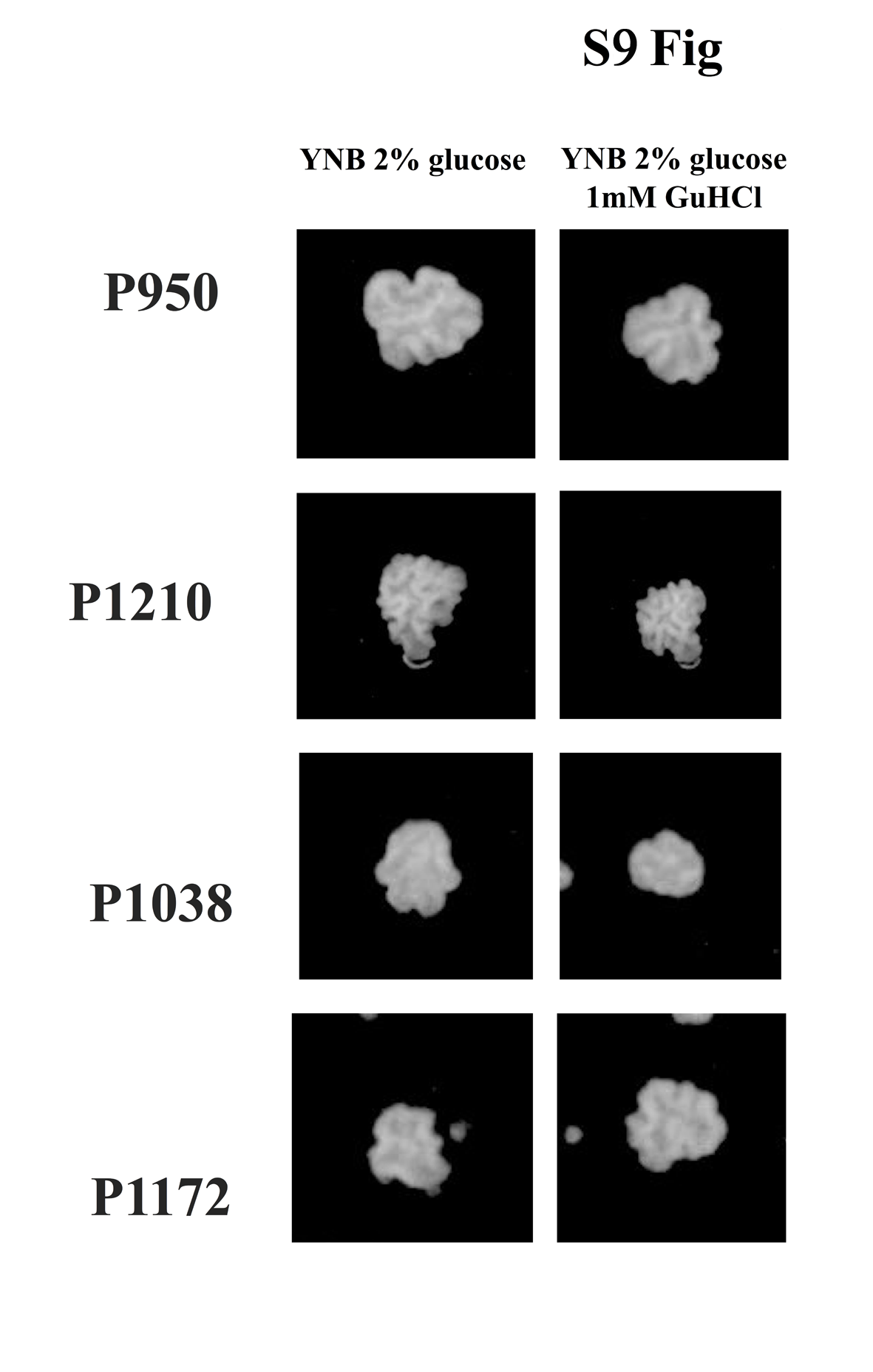

Supplement: S9 Fig — (TIF) [file pone.0161741.s009.tif]
