# Supplementary material for: Novel Centromeric Loci of the Wine and Beer Yeast Dekkera bruxellensis CEN1 and CEN2
Source: PLoS One. 2016 Aug 25;11(8):e0161741. doi: 10.1371/journal.pone.0161741 (PMC4999066; doi:10.1371/journal.pone.0161741)
Supplement: S1 Materials and Methods — Development of LAC4 reporter system and β-galactosidase assay. Deletion studies of CEN2. (DOCX) [file pone.0161741.s010.docx]

# Supporting Information

# S1 Materials and Methods

## Plasmids and molecular-biology techniques

All plasmids constructed in this study are listed in Table 2. The plasmid P935 (S4 Fig) was constructed on the base of P892 [2]. The β-galactosidase gene *LAC4* was amplified from the genomic DNA of *Kluyveromyces lactis* CBS 2359 (Y1376) using primers FB1 and RB7 and then digested with *Sal*I. The *D. bruxellensis* promoter belonging to the highly expressed gene *YPR100W* (*MRPL51*, mitochondrial ribosomal protein of the large subunit) was amplified using the primers IS290 and IS291. Then it was digested with *Sal*I and *Bam*HI. The promoter and *LAC4* were sub-cloned into P892, pre-digested with *Sal*I and *Bam*HI.

The plasmid P1160 (S4 Fig) was constructed on the base of P935. For this purpose the *CEN2* motif sequence was amplified from Y879 genomic DNA using the primers pair OP91 and OP92, digested with *Xba*I, and cloned into corresponding site of P935.

The P1211 (S4 Fig) was constructed on the base of P950 (Table 2), which was linearized by *Xma*I. The linearized plasmid was ligated to fragment carrying *K. lactis LAC4* fused with the *D. bruxellensis* promoter *YPR100W*, which was amplified from P935 with the primers OP132 and OP133 and digested with *Xma*I.

The plasmid DNA isolations from *E. coli* transformants were carried out using a GeneJET Plasmid Miniprep Kit (Thermo Fisher Scientific, Waltham, USA). All the enzymes that were used for cloning (Phusion DNA polymerase, T4 DNA ligase and restriction enzymes) were purchased from Thermo Scientific (Waltham, USA).

## Development of *LAC4* reporter system and β-galactosidase assay

To determine the *CEN* plasmids copy number, we first developed a gene reporter system for *D. bruxellensis.* The genome of *D. bruxellensis* Y879 contains one gene homologous to *K. lactis* *LAC4* (<http://genome.jgi.doe.gov/Dekbr2/Dekbr2.home.html>). However, while Y879 strain displayed β-galactosidase activity, Y997 did not (S3 Fig), and its *LAC4* gene is likely mutated. Therefore Y997 can be used as a host for *LAC4* expression. The plasmid P935 carrying *K. lactis* *LAC4* gene under control of *D. bruxellensis* promoter *YPR100W*, was digested with *Sac*I and transformed into Y997 to obtain strain Y1377, carrying integrated *LAC4*, for the expression studies (S5 Fig). This strain carried one copy of the cassette (*LAC4* and *URA3* genes) as confirmed by Southern blotting (S5 Fig) and was used further as a control strain. In addition two *CEN2* varieties (with and without the transposon: *CEN2* of Y881 and *CEN2* of Y879 respectively) sub-cloned into P935 (S4 Fig) were constructed to provide replicative reporter plasmids P1211 and P1160 (Table 2).

β-Galactosidase assays to measure the strength of the *LAC4* gene expression in *D. bruxellensis* were performed on cells permeabilized by digitonin as described previously [1].

## Deletion studies of *CEN2*

As *CEN1* did not induce chromosomal breaks but had centromere properties when cloned on the plasmid, we assumed that it is minimum sequence important for the function and did not proceed for its further sub-cloning. As *CEN2* induced chromosomal breaks we performed its further sub-cloning. The corresponding primers (S1 Table) were used to amplify and clone the shorter parts of CEN2 of *D. bruxellensis* CBS 2796 (Y881 strain) (Fig 3d) into integrative plasmid P892 [2]: OP91 and OP127 for CEN2-1 (1-1921 bp - size of 1921 bp); OP93 and OP94 for CEN2-2 (1925-2120 bp - size of 196 bp); OP125 and OP126 for CEN2-3 (2121-2602 bp - size of 482 bp); OP93 and OP126 for CEN2-4 (1925-2602 bp – size of 678 bp); SW9 and SW10 for CEN2-5 (1290-2602 bp - size of 1313 bp). The transformation efficiency and replicative properties of the constructed plasmids designated P1202, P1173, P1174, P1209 and P1038, respectively (Table 2), was studied using Y997 as a host (Fig 3cd).

# References

1. Ishchuk OP, Dmytruk KV, Rohulya OV, Voronovsky AY, Abbas CA, Sibirny AAA. Development of a promoter assay system for the flavinogenic yeast *Candida famata* based on the *Kluyveromyces lactis* β-galactosidase *LAC4* reporter gene. Enzyme Microb Technol. 2008;42(3): 208-215.
2. [Schifferdecker AJ](http://www.ncbi.nlm.nih.gov/pubmed/?term=Schifferdecker%20AJ%5BAuthor%5D&cauthor=true&cauthor_uid=26743658), [Siurkus J](http://www.ncbi.nlm.nih.gov/pubmed/?term=Siurkus%20J%5BAuthor%5D&cauthor=true&cauthor_uid=26743658), [Andersen MR](http://www.ncbi.nlm.nih.gov/pubmed/?term=Andersen%20MR%5BAuthor%5D&cauthor=true&cauthor_uid=26743658), [Joerck-Ramberg D](http://www.ncbi.nlm.nih.gov/pubmed/?term=Joerck-Ramberg%20D%5BAuthor%5D&cauthor=true&cauthor_uid=26743658), [Ling Z](http://www.ncbi.nlm.nih.gov/pubmed/?term=Ling%20Z%5BAuthor%5D&cauthor=true&cauthor_uid=26743658), [Zhou N](http://www.ncbi.nlm.nih.gov/pubmed/?term=Zhou%20N%5BAuthor%5D&cauthor=true&cauthor_uid=26743658), et al. Alcohol dehydrogenase gene *ADH3* activates glucose alcoholic fermentation in genetically engineered *Dekkera bruxellensis* yeast. Appl Microbiol Biotechnol. 2016;100(7): 3219-3231.
