# Supplementary material for: Novel Centromeric Loci of the Wine and Beer Yeast Dekkera bruxellensis CEN1 and CEN2
Source: PLoS One. 2016 Aug 25;11(8):e0161741. doi: 10.1371/journal.pone.0161741 (PMC4999066; doi:10.1371/journal.pone.0161741)
Supplement: S1 Table — (DOCX) [file pone.0161741.s011.docx]

**S1 Table. Oligonucleotides used in this study.**

| **Name** | Sequence 5’-3’ |
| --- | --- |
| FB1 | CGCGTCGACATGTCTTGCCTTATTCCTG |
| RB7 | CGCGTCGACAAGGAACATGTAGTGAAAATC |
| IS290 | CGTGGATCCACGTTATGAAGAGTTCATTCAG |
| IS291 | GAGGTCGACCACTATGTGCCAAACGTTGATTATG |
| SW9 | GCGTCTAGAAACAGAGAGGCCTTGAGTAG |
| SW10 | TTTGGATCCCTGAGGTTGCTAAGCCCC |
| OP44 | GTGAAAGTGAAGCCAACTGGTATCTAAAGAGATAC |
| OP45 | CTGAGGTTGCTAAGCCCCAAAAATAACTTCG |
| OP91 | TTATCTAGAGTGAAAGTGAAGCCAACTGGTATCTAAAGAG |
| OP92 | ATATCTAGACTGAGGTTGCTAAGCCCCAAAAATAACTTC |
| OP93 | CCCTCTAGATGTCAAAACAATGTAGCTCTCGAAC |
| OP94 | CGCGTCTAGATGTTAAAGTTAGTGTTACCAGATTTATATAG |
| OP98 | CTATCTAGAGATCTTTGCCTGCTGTTGCTTAATAATTG |
| OP99 | TTTTCTAGAGTTGGTGACAATTTGTCCGAGTGG |
| OP125 | CGCTCTAGACATCGAAATTCTGCTCCACTC |
| OP126 | TTTTCTAGACTGAGGTTGCTAAGCCCCAAAAATAAC |
| OP127 | CAATCTAGATGTCGGGAAGAGTTAGACTTATGG |
| OP132 | TTTCCCGGGACGTTATGAAGAGTTCATTCAG |
| OP133 | TCGCCCGGGAAGGAACATGTAGTGAAAATC |
| URA3-1 | CAAATGCACATGGTGTTACTGGACCTGG |
| URA3-2 | CATCCAAGCCAACACCAGGTGTCAAAATAAG |
| ORF085-1 | CACACAACGCTTGAAAACGCTGATTGCAC |
| ORF085-2 | GGACAACACTGGAGCGTAGGAAACCAATG |
