# Supplementary material for: Novel Centromeric Loci of the Wine and Beer Yeast Dekkera bruxellensis CEN1 and CEN2
Source: PLoS One. 2016 Aug 25;11(8):e0161741. doi: 10.1371/journal.pone.0161741 (PMC4999066; doi:10.1371/journal.pone.0161741)
Supplement: S2 Table — (DOCX) [file pone.0161741.s012.docx]

**S2 Table. The frequency of appearance of *CEN* loci in the *D. bruxellensis* CBS 2499 (Y879) genome (**[**http://genome.jgi.doe.gov/Dekbr2/Dekbr2.home.html**](http://genome.jgi.doe.gov/Dekbr2/Dekbr2.home.html)**).**

| ***CEN* locus** | **Number of alignment hits^#^** | **Scaffold #** |
| --- | --- | --- |
| *CEN1* | 3 | 1, 4 |
| *CEN2-1* | 1 | 6 |
| *CEN2-2* | 51 | 1, 2, 3, 4, 5, 6, 7, 8, 9, 10, 11, 14, 16, 17, 18, 21, 47, 36 |
| *CEN2-3* | 1 | 6 |
| *CEN2-4* | 52 | 1, 2, 3, 4, 5, 6, 7, 8, 9, 10, 11, 14, 16, 17, 18, 21, 47, 36 |
| *CEN2-5* | 52 | 1, 2, 3, 4, 5, 6, 7, 8, 9, 10, 11, 14, 16, 17, 18, 21, 47, 36 |

^#^ - The E-value < 1e-10 as a parameter to filter the Blast results followed by another local script, which can filter the hits shorter than ⅓ of the total length of the query.
