# Supplementary material for: Novel Centromeric Loci of the Wine and Beer Yeast Dekkera bruxellensis CEN1 and CEN2
Source: PLoS One. 2016 Aug 25;11(8):e0161741. doi: 10.1371/journal.pone.0161741 (PMC4999066; doi:10.1371/journal.pone.0161741)
Supplement: S3 Table — (DOCX) [file pone.0161741.s013.docx]

**S3 Table. Motifs found in *D. bruxellensis CEN1* and *CEN2.***

| **Name** | **Centromere properties on the plasmid** | **Association with CenH3** | **Induction of chromosomal breaks** | **Size, kb** | **AT content, %** | **Motifs found** | **Motifs** | **Position, bp** |
| --- | --- | --- | --- | --- | --- | --- | --- | --- |
| *CEN1* | + | + | - | 0.917 | 65.6 | CDEI-like (Sc) | TCA*G*GTG | 88..94 |
|  |  |  |  |  |  |  | ATCAGGTG | 87..94 |
|  |  |  |  |  |  |  | TC*T*CGTG | 519..525 |
|  |  |  |  |  |  | CDEI-like (Nc) | GGGTAA | 646..651 |
|  |  |  |  |  |  | CDEII-like (Nc) | ACGGTTAT | 755..762 |
|  |  |  |  |  |  | CDEIII-like (Sc) | TC*T*GAA | 48..53 |
|  |  |  |  |  |  |  | *C*CCGAA | 583..588 |
|  |  |  |  |  |  |  | TCC*T*AA | 693..698 |
|  |  |  |  |  |  |  | T*T*CGAA | 788..793 |
|  |  |  |  |  |  |  |  | 844..849 |
|  |  |  |  |  |  |  | TCCGA*G* | 143..138 |
|  |  |  |  |  |  | CDEIII-like (Nc) | TCCT | 887..890 |
|  |  |  |  |  |  | DbMOTIF1 | ACTTGGAG | 386..393 |
|  |  |  |  |  |  | DbMOTIF4 | TACTTGAA | 205..198 |
|  |  |  |  |  |  | ARS core *Sc*-like, 9 bp match | TGTTGTGTTTA | 153..163 |
|  |  |  |  |  |  |  | GTTTATATTTC | 253..263 |
|  |  |  |  |  |  |  | ATTTCAATTTA | 259..269 |
|  |  |  |  |  |  |  | GGTTATATTTT | 284..294 |
|  |  |  |  |  |  |  | TTATATTTTTA | 286..296 |
|  |  |  |  |  |  |  | GTTTATATGTT | 350..360 |
|  |  |  |  |  |  |  | TTATATGTTAT | 352..362 |
|  |  |  |  |  |  | ARS core *Ca*-like, 9 bp match | TTATATGTTAT | 352..362 |
|  |  |  |  |  |  | ARS core *Pg*-like, 10 bp match | AAATTAAAAAA | 811..821 |
|  |  |  |  |  |  | ARS core *Yl*-like, 12 bp match | TACGTTATGGATAG | 106..119 |
|  |  |  |  |  |  | ARS box *Sc*-like, 6 bp match | TCTGAA | 48..53 |
|  |  |  |  |  |  |  | TGTAAA | 82..87 |
|  |  |  |  |  |  |  | TTTAAA | 160..165 |
|  |  |  |  |  |  |  |  | 222..227 |
|  |  |  |  |  |  |  | TTTGAA | 209..214 |
|  |  |  |  |  |  |  | TATAAA | 734..739 |
|  |  |  |  |  |  |  |  | 746..751 |
|  |  |  |  |  |  |  | TTCATA | 575..570 |
|  |  |  |  |  |  |  | TTTAGA | 293..298 |
|  |  |  |  |  |  |  | TTCAAA | 324..329 |
|  |  |  |  |  |  |  |  | 394..399 |
|  |  |  |  |  |  |  |  | 566..571 |
|  |  |  |  |  |  |  | TTCACA | 213..218 |
|  |  |  |  |  |  |  | TTCAGA | 521..516 |
|  |  |  |  |  |  |  | TATGAA | 389..384 |
|  |  |  |  |  |  | ARS box *Ca*-like, 6 bp match | ATATGT | 578..573 |
|  |  |  |  |  |  |  | TTTGAA | 209..214 |
|  |  |  |  |  |  |  | TTCAAA | 324..329 |
|  |  |  |  |  |  |  |  | 394..399 |
|  |  |  |  |  |  |  |  | 566..571 |
|  |  |  |  |  |  | *Y. lypolitica* CB4-like | ATACGTATTTCTA | 763..775 |
|  |  |  |  |  |  | *Y. lypolitica* CB5-like | ATGGATAGCGT*T* | 112..123 |
|  |  |  |  |  |  |  | AAGTATACAACC | 570..581 |
|  |  |  |  |  |  |  | TTTTCAAAGATA | 322..333 |
|  |  |  |  |  |  | *Y. lypolitica* CB6-like | AAAATAGTTATAA | 738..750 |
|  |  |  |  |  |  |  | TTATATGTTATTT | 352..364 |
|  |  |  |  |  |  | Ser tRNA gene | CGACAACTGCAGGATTCGAACCTGCGCGGGCAGAGCCCAAAAGATTTCTAATCTTTCTCCTTAACCACTCGG | 830..901 |
|  |  |  |  |  |  | HELITRON11_CB-like | CGCAGGTTCGAATCCTGCAGTTGTCGGCTTATCTTTTTTTAATTTT | 855..810 |
| *CEN2-5* | + | + | - | 1.313 | 67.0 | CDEI-like (Sc) | TCACA*C*G | 476..482 |
|  |  |  |  |  |  | CDEIII-like (Sc) | TC*G*GAA | 78..83 |
|  |  |  |  |  |  |  |  | 1178..1183 |
|  |  |  |  |  |  |  | *A*CCGAA | 1108..1113 |
|  |  |  |  |  |  |  | TC*T*GAA | 463..468 |
|  |  |  |  |  |  |  | TCC*A*AA | 871..876 |
|  |  |  |  |  |  |  | *C*CCGAA | 248..243 |
|  |  |  |  |  |  |  | TCC*T*AA | 90..95 |
|  |  |  |  |  |  |  | TCCG*G*A | 108..113 |
|  |  |  |  |  |  | DbMOTIF1 | ACTTGGAG | 453..446 |
|  |  |  |  |  |  | DbMOTIF4 | TACTTGAA | 674..668 |
|  |  |  |  |  |  | ARS core *Sc*-like, 9 bp match | TATTCTATTTA | 306..316 |
|  |  |  |  |  |  |  | TTATTTATTTT | 853..863 |
|  |  |  |  |  |  |  | TTTTACACTTT | 861..871 |
|  |  |  |  |  |  |  | ATTTAAAGTTT | 900..910 |
|  |  |  |  |  |  |  | TTTAAAGTTTT | 901..911 |
|  |  |  |  |  |  |  | TACATACAAAA | 1142..1152 |
|  |  |  |  |  |  |  | TGAACAAAAAT | 939..949 |
|  |  |  |  |  |  |  | ACTATATAAAT | 800..810 |
|  |  |  |  |  |  |  | TAAACATACAC | 595..605 |
|  |  |  |  |  |  |  | TGATTATAAAT | 289..299 |
|  |  |  |  |  |  |  | GAAATATAGAA | 221..231 |
|  |  |  |  |  |  |  | TAACAATAAAA | 22..32 |
|  |  |  |  |  |  | ARS core *Sc*-like, 10 bp match | ATTTATTTTTA | 855..865 |
|  |  |  |  |  |  |  | TAAATATAAAC | 295..305 |
|  |  |  |  |  |  | ARS core *Ca*-like, 9 bp match | TTATTTATTTT | 853..863 |
|  |  |  |  |  |  |  | TTTTGAATTAT | 925..935 |
|  |  |  |  |  |  |  | AGGATACAAAA | 1162..1172 |
|  |  |  |  |  |  |  | TACATACAAAA | 1142..1152 |
|  |  |  |  |  |  |  | AAAAGTCAAAA | 1037..1047 |
|  |  |  |  |  |  |  | TTTAAAGTTTT | 901..1001 |
|  |  |  |  |  |  | ARS core *Pg*-like, 9 bp match | AAATCAAACAG | 946..956 |
|  |  |  |  |  |  |  | ATTTATTTTTA | 855..865 |
|  |  |  |  |  |  | ARS core *Pg*-like, 10 bp match | ATTTATCTGAA | 458..468 |
|  |  |  |  |  |  | ARS box *Sc*-like, 6 bp match | TCTGAA | 463..468 |
|  |  |  |  |  |  |  | TTTAAA | 428..433 |
|  |  |  |  |  |  |  |  | 901..906 |
|  |  |  |  |  |  |  | TTTGAA | 926..931 |
|  |  |  |  |  |  |  | TATAAA | 293..298 |
|  |  |  |  |  |  |  |  | 299..304 |
|  |  |  |  |  |  |  |  | 726..731 |
|  |  |  |  |  |  |  |  | 804..809 |
|  |  |  |  |  |  |  |  | 1021..1026 |
|  |  |  |  |  |  |  | TTTAGA | 1258..1263 |
|  |  |  |  |  |  |  | TTCAAA | 450..455  1051..1056 |
|  |  |  |  |  |  |  | TTCACA | 1119..1114 |
|  |  |  |  |  |  |  |  | 475..480 |
|  |  |  |  |  |  |  | TTTACA | 399..394 |
|  |  |  |  |  |  |  |  | 553..558 |
|  |  |  |  |  |  |  |  | 862..867 |
|  |  |  |  |  |  |  |  | 1010..1015 |
|  |  |  |  |  |  |  | TTCAGA | 618..613 |
|  |  |  |  |  |  |  | TCTGAA | 463..468 |
|  |  |  |  |  |  |  | TATGAA | 380..385 |
|  |  |  |  |  |  | ARS box *Ca*-like, 6 bp match | ATATGT | 203..208 |
|  |  |  |  |  |  |  |  | 587..582 |
|  |  |  |  |  |  |  |  | 764..759 |
|  |  |  |  |  |  |  |  | 1083..1078 |
|  |  |  |  |  |  |  | TACACA | 345..350 |
|  |  |  |  |  |  |  |  | 1012..1017 |
|  |  |  |  |  |  |  | TTTGAA | 926..931 |
|  |  |  |  |  |  |  |  | 1011..1006 |
|  |  |  |  |  |  |  | TTCAAA | 450..455 |
|  |  |  |  |  |  |  |  | 1051..1056 |
|  |  |  |  |  |  |  |  | 910..905 |
|  |  |  |  |  |  |  | ACATAT | 172..167 |
|  |  |  |  |  |  | *Y. lypolitica* CB1-like | ATCGTGTCA | 632..640 |
|  |  |  |  |  |  | *Y. lypolitica* CB2-like | GGACATGAA | 973..981 |
|  |  |  |  |  |  | *Y. lypolitica* CB5-like | TACACTTTCCAT | 601..612 |
|  |  |  |  |  |  |  | CATACTATCTAT | 763..774 |
|  |  |  |  |  |  |  | ATACATACAAAA | 1141..1152 |
|  |  |  |  |  |  | *Y. lypolitica* CB6-like | CTAATTGATATTA | 92..104 |
|  |  |  |  |  |  | Transib-14_HM-like | ATTTTGTTTACTTTCTTTACTGAGTAAATAGAATAGTTTATATTTATAATCAGCTTTTCTTGTTCACATAACACCAAAGCTATAGATATAAT | 340..249 |
|  |  |  |  |  |  | HAL1-1E_Tbel-like | TTGTTCATTAATAATTCAAAACTCGCATTATGCAAAAACTTTAAATTTGATTTTGAGATACGAACAATTTTTGGAAAGTGTAAAAATAAATAA | 945..853 |
|  |  |  |  |  |  | L1-95_ACar-like | ACATGAAATTAAAGAGACAAGGAATTCAATTAAACTTTACACACCTTATAAAATCAATAGTGAAAA | 975..1040 |
|  |  |  |  |  |  | L1-71_ACar-like | TCAAAATTGTTCAAAAGAAAATTGAGTACATCAAGCTATACAAGAA | 1042..1087 |
|  |  |  |  |  |  | Polinton-3_HM-like | GATACATACAAAAAAGGAATTTAGGATACAAAAATATTT | 1178..1140 |
|  |  |  |  |  |  | Transposon *CEN2-2* | TGTCAAAACAATGTAGCTCTCGAACAATGGAGTTCAAGTAAGTGAAGTTGGTATTGTTATATCATCTGATCAATACGAGAACCACATCTATATAAAGTGAAGATATTCCCTTTTGTCTATCATTATACATACTATCTATTACATACTACAATAAATATCGAAACACTATATAAATCTGGTAACACTAACTTTAACA | 1925..2120 |
| *CEN2* Y881 | + | + (part amplified with SW9/SW10 primers) | + | 2.602 | 63.3 | CDEI-like (Sc) | TCACA*C*G | 1765..1771 |
|  |  |  |  |  |  |  | *G*CACATG | 413..419 |
|  |  |  |  |  |  | CDEI-like (Nc) | TTACCC | 996..1001 |
|  |  |  |  |  |  | CDEIII-like (Sc) | TC*G*GAA | 105..110 |
|  |  |  |  |  |  |  |  | 1367..1372 |
|  |  |  |  |  |  |  |  | 2467..2472 |
|  |  |  |  |  |  |  | T*G*CGAA | 114..119 |
|  |  |  |  |  |  |  | *A*CCGAA | 230..235 |
|  |  |  |  |  |  |  |  | 1128..1133 |
|  |  |  |  |  |  |  |  | 2397..2402 |
|  |  |  |  |  |  |  | TC*T*GAA | 276..281 |
|  |  |  |  |  |  |  |  | 1752..1757 |
|  |  |  |  |  |  |  | TCC*A*AA | 801..806 |
|  |  |  |  |  |  |  |  | 2160..2165 |
|  |  |  |  |  |  |  | *C*CCGAA | 999..1004 |
|  |  |  |  |  |  |  |  | 1537..1532 |
|  |  |  |  |  |  |  | TCCGA*G* | 1050..1055 |
|  |  |  |  |  |  |  | TC*A*GAA | 1086..1091 |
|  |  |  |  |  |  |  |  | 1185..1190 |
|  |  |  |  |  |  |  | TCC*T*AA | 1379..1384 |
|  |  |  |  |  |  |  | TCCG*G*A | 1397..1402 |
|  |  |  |  |  |  | DbMOTIF1 | ACTTGGAG | 1742..1735 |
|  |  |  |  |  |  | DbMOTIF4 | TACTTGAA | 1203..1210 |
|  |  |  |  |  |  |  |  | 1964..1957 |
|  |  |  |  |  |  | ARS core *Sc*-like, 9 bp match | TTTTATGATTG | 1213..1223 |
|  |  |  |  |  |  |  | TATTCTATTTA | 1595..1605 |
|  |  |  |  |  |  |  | TTATTTATTTT | 2142..2152 |
|  |  |  |  |  |  |  | TTTTACACTTT | 2150..2160 |
|  |  |  |  |  |  |  | ATTTAAAGTTT | 2189..2199 |
|  |  |  |  |  |  |  | TTTAAAGTTTT | 2190..2200 |
|  |  |  |  |  |  |  | TACATACAAAA | 2431..2441 |
|  |  |  |  |  |  |  | TGAACAAAAAT | 2228..2238 |
|  |  |  |  |  |  |  | ACTATATAAAT | 2089..2099 |
|  |  |  |  |  |  |  | TAAACATACAC | 1884..1894 |
|  |  |  |  |  |  |  | TGATTATAAAT | 1578..1588 |
|  |  |  |  |  |  |  | GAAATATAGAA | 1510..1520 |
|  |  |  |  |  |  |  | TAACAATAAAA | 1311..1321 |
|  |  |  |  |  |  |  | AAAATGTATAA | 978..988 |
|  |  |  |  |  |  | ARS core *Sc*-like, 10 bp match | ATTTATTTTTA | 2144..2154 |
|  |  |  |  |  |  |  | TAAATATAAAC | 1584..1594 |
|  |  |  |  |  |  | ARS core *Ca*-like, 9 bp match | TTATTTATTTT | 2142..2152 |
|  |  |  |  |  |  |  | TTTTGAATTAT | 2214..2224 |
|  |  |  |  |  |  |  | AGGATACAAAA | 2451..2461 |
|  |  |  |  |  |  |  | TACATACAAAA | 2431..2441 |
|  |  |  |  |  |  |  | AAAAGTCAAAA | 2326..2336 |
|  |  |  |  |  |  |  | AAAGTACCAAA | 683..693 |
|  |  |  |  |  |  |  | GAAATGCAAAA | 175..185 |
|  |  |  |  |  |  |  | TTTTATGATTG | 1213..1223 |
|  |  |  |  |  |  |  | TTTAAAGTTTT | 2190..2200 |
|  |  |  |  |  |  | ARS core *Pg*-like, 9 bp match | GAATCAGAAAA | 1182..1192 |
|  |  |  |  |  |  |  | AAATCAAACAG | 2235..2245 |
|  |  |  |  |  |  |  | TTTTATGATTG | 1213..1223 |
|  |  |  |  |  |  |  | AAGAGATACAT | 27..37 |
|  |  |  |  |  |  |  | ATTTATTTTTA | 2144..2154 |
|  |  |  |  |  |  | ARS core *Pg*-like, 10 bp match | ATTTATCTGAA | 1747..1757 |
|  |  |  |  |  |  | ARS box *Sc*-like, 6 bp match | TCTGAA | 276..281 |
|  |  |  |  |  |  |  |  | 1752..1757 |
|  |  |  |  |  |  |  | TTTAAA | 1717..1722 |
|  |  |  |  |  |  |  |  | 2190..2195 |
|  |  |  |  |  |  |  | TTTGAA | 567..572 |
|  |  |  |  |  |  |  |  | 2215..2220 |
|  |  |  |  |  |  |  | TATAAA | 1582..1587 |
|  |  |  |  |  |  |  |  | 1588..1593 |
|  |  |  |  |  |  |  |  | 2015..2022 |
|  |  |  |  |  |  |  |  | 2093..2098 |
|  |  |  |  |  |  |  |  | 2310..2315 |
|  |  |  |  |  |  |  | TTCATA | 906..911 |
|  |  |  |  |  |  |  | TTTAGA | 1168..1173 |
|  |  |  |  |  |  |  |  | 2547..2552 |
|  |  |  |  |  |  |  | TTCAAA | 975..980 |
|  |  |  |  |  |  |  |  | 1739..1744 |
|  |  |  |  |  |  |  |  | 2340..2345 |
|  |  |  |  |  |  |  | TTCACA | 196..191 |
|  |  |  |  |  |  |  |  | 799..794 |
|  |  |  |  |  |  |  |  | 2408..2403 |
|  |  |  |  |  |  |  |  | 345..350 |
|  |  |  |  |  |  |  |  | 1764..1769 |
|  |  |  |  |  |  |  | TTCACA | 345..350 |
|  |  |  |  |  |  |  |  | 1764..1769 |
|  |  |  |  |  |  |  | TTTACA | 1842..1847 |
|  |  |  |  |  |  |  |  | 2151..2156 |
|  |  |  |  |  |  |  |  | 2299..2304 |
|  |  |  |  |  |  |  |  | 697..692 |
|  |  |  |  |  |  |  |  | 952..947 |
|  |  |  |  |  |  |  |  | 984..979 |
|  |  |  |  |  |  |  | TTCAGA | 1907..1902 |
|  |  |  |  |  |  |  | TCTAAA | 23..28 |
|  |  |  |  |  |  |  |  | 1487..1482 |
|  |  |  |  |  |  |  | TATGAA | 1669..1674 |
|  |  |  |  |  |  | ARS box *Ca*-like, 6 bp match | ATATGT | 1876..1871 |
|  |  |  |  |  |  |  |  | 2053..2048 |
|  |  |  |  |  |  |  |  | 2372..2367 |
|  |  |  |  |  |  |  |  | 391..396 |
|  |  |  |  |  |  |  |  | 1492..1497 |
|  |  |  |  |  |  |  | TACACA | 1634..1639  2301..2306 |
|  |  |  |  |  |  |  | TTTGAA | 567..572 |
|  |  |  |  |  |  |  |  | 2215..2220 |
|  |  |  |  |  |  |  |  | 544..539 |
|  |  |  |  |  |  |  |  | 2300..2295 |
|  |  |  |  |  |  |  | TTCAAA | 975..980 |
|  |  |  |  |  |  |  |  | 1739..1744 |
|  |  |  |  |  |  |  |  | 2340..2345 |
|  |  |  |  |  |  |  |  | 1011..1006 |
|  |  |  |  |  |  |  |  | 2199..2194 |
|  |  |  |  |  |  |  | ACATAT | 149..154 |
|  |  |  |  |  |  |  |  | 987..982 |
|  |  |  |  |  |  |  |  | 1461..1456 |
|  |  |  |  |  |  | *Y. lypolitica* CB1-like | ATCGTGTCA | 1921..1929 |
|  |  |  |  |  |  | *Y. lypolitica* CB2-like | AAAATGTCA | 691..699 |
|  |  |  |  |  |  |  | GGACATGAA | 2262..2270 |
|  |  |  |  |  |  |  | CAAATTTCA | 1155..1163 |
|  |  |  |  |  |  |  | GAGAAGTCC | 59..67 |
|  |  |  |  |  |  | *Y. lypolitica* CB5-like | TACACTTTCCAT | 1890..1901 |
|  |  |  |  |  |  |  | CATACTATCTAT | 2052..2063 |
|  |  |  |  |  |  |  | ATACATACAAAA | 2430..2441 |
|  |  |  |  |  |  |  | TATGATATGCAT | 80..91 |
|  |  |  |  |  |  | *Y. lypolitica* CB6-like | CTAATTGATATTA | 1381..1393 |
|  |  |  |  |  |  | Harbinger-10_PSt-like | GATTTAGTACTTGAAGGTTTTATGATTGCATAGAAATCCAAT | 1196..1237 |
|  |  |  |  |  |  | Transib-14_HM-like | ATTTTGTTTACTTTCTTTACTGAGTAAATAGAATAGTTTATATTTATAATCAGCTTTTCTTGTTCACATAACACCAAAGCTATAGATATAAT | 1629..1538 |
|  |  |  |  |  |  | HAL1-1E_Tbel-like | TTGTTCATTAATAATTCAAAACTCGCATTATGCAAAAACTTTAAATTTGATTTTGAGATACGAACAATTTTTGGAAAGTGTAAAAATAAATAA | 2234..2142 |
|  |  |  |  |  |  | L1-95_ACar-like | ACATGAAATTAAAGAGACAAGGAATTCAATTAAACTTTACACACCTTATAAAATCAATAGTGAAAA | 2264..2329 |
|  |  |  |  |  |  | L1-71_ACar-like | TCAAAATTGTTCAAAAGAAAATTGAGTACATCAAGCTATACAAGAA | 2331..2376 |
|  |  |  |  |  |  | Polinton-3_HM-like | AAATATTTTTGTATCCTAAATTCCTTTTTTGTATGTATC | 2467..2429 |
|  |  |  |  |  |  | Transposon *CEN2-2* | TGTCAAAACAATGTAGCTCTCGAACAATGGAGTTCAAGTAAGTGAAGTTGGTATTGTTATATCATCTGATCAATACGAGAACCACATCTATATAAAGTGAAGATATTCCCTTTTGTCTATCATTATACATACTATCTATTACATACTACAATAAATATCGAAACACTATATAAATCTGGTAACACTAACTTTAACA | 1925..2120 |
| *CEN2* Y879 | + | + (part amplified with SW9/SW10 primers) | + | 2.352 | 63.1 | CDEI-like (Sc) | *G*CACATG | 414..420 |
|  |  |  |  |  |  | CDEI-like (Nc) | GGGTAA | 1800..1805 |
|  |  |  |  |  |  |  | TTACCC | 996..1001 |
|  |  |  |  |  |  | CDEIII-like (Sc) | TC*G*GAA | 106..111 |
|  |  |  |  |  |  |  |  | 1367..1372 |
|  |  |  |  |  |  |  |  | 2266..2271 |
|  |  |  |  |  |  |  | T*G*CGAA | 115..120 |
|  |  |  |  |  |  |  | *A*CCGAA | 1128..1133 |
|  |  |  |  |  |  |  |  | 2196..2201 |
|  |  |  |  |  |  |  | TC*T*GAA | 277..282 |
|  |  |  |  |  |  |  |  | 1751..1756 |
|  |  |  |  |  |  |  | TCC*A*AA | 802..807 |
|  |  |  |  |  |  |  | *C*CCGAA | 999..1004 |
|  |  |  |  |  |  |  |  | 1536..1531 |
|  |  |  |  |  |  |  | TC*A*GAA | 1086..1091 |
|  |  |  |  |  |  |  |  | 1185..1190 |
|  |  |  |  |  |  |  | TCC*T*AA | 1379..1384 |
|  |  |  |  |  |  |  | TCCG*G*A | 1397..1402 |
|  |  |  |  |  |  | DbMOTIF1 | ACTTGGAG | 1734..1741 |
|  |  |  |  |  |  | DbMOTIF4 | TACTTGAA | 1203..1210 |
|  |  |  |  |  |  | ARS core *Sc*-like, 9 bp match | TTTTATGATTG | 1213..1223 |
|  |  |  |  |  |  |  | TATTCTATTTA | 1594..1603 |
|  |  |  |  |  |  |  | TTATTTATTTT | 1941..1951 |
|  |  |  |  |  |  |  | ATTTAAAGTTT | 1988..1998 |
|  |  |  |  |  |  |  | TTTAAAGTTTT | 1989..1999 |
|  |  |  |  |  |  |  | TAAACATACAC | 1884..1894 |
|  |  |  |  |  |  |  | GAAATATAGAA | 1509..1519 |
|  |  |  |  |  |  |  | TAACAATAAAA | 1311..1321 |
|  |  |  |  |  |  |  | AAAATGTATAA | 979..989 |
|  |  |  |  |  |  | ARS core *Sc*-like, 10 bp match | ATTTATTTTTA | 1943..1953 |
|  |  |  |  |  |  | ARS core *Ca*-like, 9 bp match | TTATTTATTTT | 1941..1951 |
|  |  |  |  |  |  | ARS core *Ca*-like, 9 bp match | TTTTGAATTAT | 2013..2023 |
|  |  |  |  |  |  |  | AGGATACAAAA | 2250..2260 |
|  |  |  |  |  |  |  | AAAAGTCAAAA | 2125..2135 |
|  |  |  |  |  |  |  | AAAGTACCAAA | 684..694 |
|  |  |  |  |  |  |  | GAAATGCAAAA | 176..186 |
|  |  |  |  |  |  |  | TTTTATGATTG | 1213..1223 |
|  |  |  |  |  |  |  | TTTAAAGTTTT | 1989..1999 |
|  |  |  |  |  |  | ARS core *Pg*-like, 9 bp match | GAATCAGAAAA | 1182..1192 |
|  |  |  |  |  |  |  | TTTTATGATTG | 1213..1223 |
|  |  |  |  |  |  |  | AAGAGATACAT | 28..38 |
|  |  |  |  |  |  |  | ATTTATTTTTA | 1943..1953 |
|  |  |  |  |  |  | ARS core *Pg*-like, 10 bp match | ATTTATCTGAA | 1746..1756 |
|  |  |  |  |  |  | ARS box *Sc*-like, 6 bp match | TCTGAA | 277..282 |
|  |  |  |  |  |  |  |  | 1751..1756 |
|  |  |  |  |  |  |  | TTTAAA | 1716..1721 |
|  |  |  |  |  |  |  |  | 1989..1994 |
|  |  |  |  |  |  |  | TTTGAA | 568..573 |
|  |  |  |  |  |  |  |  | 2014..2019 |
|  |  |  |  |  |  |  | TATAAA | 1587..1592 |
|  |  |  |  |  |  |  |  | 2109..2114 |
|  |  |  |  |  |  |  | TTTAGA | 2346..2351 |
|  |  |  |  |  |  |  | TTCAAA | 976..981 |
|  |  |  |  |  |  |  |  | 1738..1743 |
|  |  |  |  |  |  |  |  | 2139..2144 |
|  |  |  |  |  |  |  | TTCACA | 197..192 |
|  |  |  |  |  |  |  |  | 800..725 |
|  |  |  |  |  |  |  |  | 346..351 |
|  |  |  |  |  |  |  | TTTACA | 1950..1955 |
|  |  |  |  |  |  |  |  | 2098..2103 |
|  |  |  |  |  |  |  |  | 698..693 |
|  |  |  |  |  |  |  |  | 953..948 |
|  |  |  |  |  |  |  |  | 985..980 |
|  |  |  |  |  |  |  |  | 1687..1682 |
|  |  |  |  |  |  |  | TTCAGA | 1862..1857 |
|  |  |  |  |  |  |  |  | 1907..1902 |
|  |  |  |  |  |  |  | TCTAAA | 24..29 |
|  |  |  |  |  |  |  |  | 1959..1964 |
|  |  |  |  |  |  |  |  | 1991..1986 |
|  |  |  |  |  |  |  | TATGAA | 1668..1673 |
|  |  |  |  |  |  | ARS box *Ca*-like, 6 bp match | ATATGT | 1876..1871 |
|  |  |  |  |  |  |  |  | 2171..2166 |
|  |  |  |  |  |  |  | TACACA | 1633..1638 |
|  |  |  |  |  |  |  |  | 2100..2105 |
|  |  |  |  |  |  |  | TTTGAA | 545..540 |
|  |  |  |  |  |  |  |  | 2099..2094 |
|  |  |  |  |  |  |  |  | 568..573 |
|  |  |  |  |  |  |  |  | 2014..2019 |
|  |  |  |  |  |  |  | TTCAAA | 976..981 |
|  |  |  |  |  |  |  |  | 1738..1743 |
|  |  |  |  |  |  |  |  | 2139..2144 |
|  |  |  |  |  |  |  |  | 1011..1006 |
|  |  |  |  |  |  |  |  | 1998..1993 |
|  |  |  |  |  |  |  | ACATAT | 150..155 |
|  |  |  |  |  |  |  |  | 1953..1958 |
|  |  |  |  |  |  |  |  | 988..983 |
|  |  |  |  |  |  |  |  | 1461..1456 |
|  |  |  |  |  |  | *Y. lypolitica* CB2-like | AAAATGTCA | 692..700 |
|  |  |  |  |  |  |  | GGACATGAA | 2061..2069 |
|  |  |  |  |  |  |  | CAAATTTCA | 1155..1163 |
|  |  |  |  |  |  |  | GAGAAGTCC | 60..68 |
|  |  |  |  |  |  | *Y. lypolitica* CB5-like | TACACTTTCCAT | 1890..1901 |
|  |  |  |  |  |  | *Y. lypolitica* CB6-like | CTAATTGATATTA | 1381..1393 |
|  |  |  |  |  |  | L1-71_ACar-like | TCAAAATTGTTCAAAAGAAAATTGAGTACATCAAGCTATACAAGAA | 2130..2175 |
