# Supplementary material for: Novel Centromeric Loci of the Wine and Beer Yeast Dekkera bruxellensis CEN1 and CEN2
Source: PLoS One. 2016 Aug 25;11(8):e0161741. doi: 10.1371/journal.pone.0161741 (PMC4999066; doi:10.1371/journal.pone.0161741)
Supplement: S4 Table — (DOCX) [file pone.0161741.s014.docx]

**S4 Table. Transformation efficiency of *S. cerevisiae* Y601 with plasmids carrying *D. bruxellensis* *CEN1* and *CEN2*.**

| **Plasmid used** | **Transformation efficiency, transformants/µg of DNA** |
| --- | --- |
| P893* | 4.23 x 10^3^ |
| P891* | 0.12 x 10^3^ |
| P950 | 0 |
| P948 | 0 |
| P949 | 0 |
| P892^#^ | 0 |

- - positive controls carrying *S. cerevisiae* 2-micron ori (P893) and *ARS/CEN* elements (P891).

^#^ - negative control, vector without any yeast replicating sequences.
