# Supplementary material for: Novel Centromeric Loci of the Wine and Beer Yeast Dekkera bruxellensis CEN1 and CEN2
Source: PLoS One. 2016 Aug 25;11(8):e0161741. doi: 10.1371/journal.pone.0161741 (PMC4999066; doi:10.1371/journal.pone.0161741)
Supplement: S5 Table — (DOCX) [file pone.0161741.s015.docx]

**S5 Table. Inverted and direct repeats found in *CEN1* and *CEN2*.**

| **Locus** | **Inverted Repeat length, bp** | **Sequence and position, bp** |
| --- | --- | --- |
| *CEN1* | 9 (1 mismatch) | - 23 A*T*AATTGAT 31 - 40 T*T*TTAACTA 32 |
|  |  | - 313 CTGAA*C*TGT 322 - 348 GACTT*T*ACA 340 |
|  |  | - 820 AAAGAT*A*AG 828 - 885 TTTCTA*A*TC 877 |
|  | 10 (1 mismatch) | - 392 AGTTCAA*A*AT 401 - 487 TCAAGTT*C*TA 478 |
|  | 11 (1 mismatch) | - 217 GTTCA*T*TTAAA 227 - 319 CAAGT*C*AATTT 309 |
|  |  | - 698 ATTTT*C*ATAAG 708 - 742 TAAAA*A*TATTC 732 |
| *CEN2* | 8 | - 735 CATCTTTG 742 - 810 GTAGAAAC 803 |
|  |  | - 1353 ATTTTTAA 1360 - 1447 TAAAAATT 1440 |
|  |  | - 2074 AATAAATA 2081 - 2150 TTATTTAT 2143 |
|  |  | - 2111 AACTTTAA 2118 - 2198 TTGAAATT 2191 |
|  |  | - 2192 TAAAGTTT 2199 - 2302 ATTTCAAA 2295 |
|  | 9 (1 mismatch) | - 42 CAA*A*AATTG 50 - 141 GTT*C*TTAAC 133 |
|  |  | - 72 ACA*C*GAATT 80 - 142 TGT*T*CTTAA 134 |
|  |  | - 112 ATTGC*G*AAC 120 - 136 TAACG*G*TTG 128 |
|  |  | - 123 A*T*TATGTTG 131 - 155 T*T*ATACAAC 147 |
|  |  | - 274 CTTCT*G*AAA 282 - 338 GAAGA*G*TTT 330 |
|  |  | - 420 AACCAG*T*AT 428 - 530 TTGGTC*T*TA 522 |
|  |  | - 681 GGAAA*G*TAC 689 - 772 CCTTT*A*ATG 764 |
|  |  | - 983 G*T*ATAACAG 991 - 1052 C*C*TATTGTC 1044 |
|  |  | - 1176 CTTCAAG*A*A 1184 - 1211 GAAGTTC*A*T 1203 |
|  |  | - 1260 T*A*AAAAATT 1268 - 1360 A*A*TTTTTAA 1352 |
|  |  | - 1453 CTTT*G*TATA 1461 - 1522 GAAA*G*ATAT 1514 |
|  |  | - 1561 ATGTG*A*ACA 1569 - 1640 TACAC*A*TGT 1632 |
|  |  | - 1584 TAAATA*T*AA 1592 - 1605 ATTTAT*C*TT 1597 |
|  |  | - 1703 TAAAT*T*CTT 1711 - 1751 ATTTA*C*GAA 1743 |
|  |  | - 1836 CA*C*TGTTTT 1844 - 1937 GT*A*ACAAAA 1929 |
|  |  | - 2010 AT*C*TATATA 2018 - 2099 TA*A*ATATAT 2091 |
|  |  | - 2015 TATA*A*AGTG 2023 - 2096 ATAT*A*TCAC 2088 |
|  |  | - 2145 TT*T*ATTTTT 2153 - 2241 AA*C*TAAAAA 2233 |
|  |  | - 2297 ACTTT*A*CAC 2305 - 2331 TGAAA*A*GTG 2323 |
|  |  | - 2494 TTCG*G*TTCG 2502 - 2575 AAGC*A*AAGC 2567 |
|  |  | - 2499 TTC*G*ACAAA 2507 - 2520 AAG*G*TGTTT 2512 |
|  |  | - 2512 TTT*G*TGGAA 2520 - 2531 AAA*G*ACCTT 2523 |
|  | 10 (1 mismatch) | - 46 AATTG*A*CAAC 55 - 137 TTAAC*G*GTTG 128 |
|  |  | - 698 CATC*A*TTGGA 707 - 810 GTAG*A*AACCT 801 |
|  |  | - 1183 AATCA*G*AAAA 1192 - 1222 TTAGT*A*TTTT 1213 |
|  |  | - 1715 GCTTT*A*AACC 1724 - 1746 CGAAA*C*TTGG 1737 |
|  |  | - 2072 A*C*AATAAATA 2081 - 2152 T*T*TTATTTAT 2143 |
|  |  | - 2190 T*T*TAAAGTTT 2199 - 2304 A*C*ATTTCAAA 2295 |
|  |  | - 2292 ATTA*A*ACTTT 2301 - 2411 TAAT*G*TGAAA 2402 |
|  | 11 (1 mismatch) | - 1539 TTATAT*C*TATA 1549 - 1592 AATATA*A*ATAT 1582 |
|  | 13 (1 mismatch) | - 1804 AATTACTTTA*G*CA 1816 - 1830 TTAATGAAAT*T*GT 1818 |
| **Locus** | **Direct Repeat length, bp** | **Sequence and position, bp** |
| *CEN1* | 5 | AGGTG 90, 597 |
|  | 6 | TTTCTA 38, 176, 182, 770, 876 |
|  |  | GTTCAT 60, 94, 200, 217 |
| *CEN2* | 5 | TGCAA 179, 509, 555, 599, 645, 713, 812, 860 |
|  | 8 | TTTGGTGT 758, 1467, 1552 |
|  | 9 | TACATACTA 2050, 2064 |
|  | 10 | AACAGAGAGG 1290, 1341 |
|  | 11 | TACACTTTCCA 1890, 2153 |
|  | 12 | TGTGATTGCAAG 549, 854 |
|  | 13 | CTGAAAGAGAAAT 874, 938 |
