# Supplementary material for: Novel Centromeric Loci of the Wine and Beer Yeast Dekkera bruxellensis CEN1 and CEN2
Source: PLoS One. 2016 Aug 25;11(8):e0161741. doi: 10.1371/journal.pone.0161741 (PMC4999066; doi:10.1371/journal.pone.0161741)
Supplement: S6 Table — (DOCX) [file pone.0161741.s016.docx]

S6 Table. **Estimation of relative *URA3* gene copy number in the *D. bruxellensis* Y997 transformants by RT-PCR.**

| **Plasmid** | **Ct values, *URA3* gene** | ***URA3* gene copy number^1^** | ***URA3* gene copy number normalized by *YML085C* gene** | **Mean** | **Relative copy number** ^2^ |
| --- | --- | --- | --- | --- | --- |
| P950 | 15.2 | 7302707.42 | 2.143546925 | 2.071773463 | 2.517155663 |
|  | 15 | 8388608 | 2 |  |  |
|  | 15.3 | 6813666.95 | 1.624504793 | 1.884025859 | 2.289046773 |
|  | 15 | 8388608 | 2.143546925 |  |  |
| P1210 | 15.4 | 6357376.058 | 1.866065983 | 2.164177405 | 2.629424263 |
|  | 15.3 | 6813666.95 | 2.462288827 |  |  |
|  | 15 | 8388608 | 1.741101127 | 1.628408847 | 1.978478161 |
|  | 15.1 | 7826848.017 | 1.515716567 |  |  |
| P1038 | 14.4 | 12714752.12 | 2 | 1.757858283 | 2.135756159 |
|  | 14.9 | 8990687.442 | 1.515716567 |  |  |
|  | 14.5 | 11863283.2 | 1.866065983 | 1.866065983 | 2.26722595 |
|  | 14.6 | 11068834.62 | 1.866065983 |  |  |
| P1172 | 14.5 | 11863283.2 | 1.319507911 | 1.472006352 | 1.788452836 |
|  | 14.4 | 12714752.12 | 1.624504793 |  |  |
|  | 15.4 | 6357376.058 | 1.624504793 | 1.57011068 | 1.907647269 |
|  | 15.3 | 6813666.95 | 1.515716567 |  |  |
| Control Y1377 | 16.1 | 3913424.009 | 1.148698355 | 1.64612264 | 2 |
|  | 15.4 | 6357376.058 | 2.143546925 |  |  |
| Control Y997 | 18.5 | 741455.2002 | 1.148698355 | 0.953278319 | 1.158210568 |
|  | 18.8 | 602248.7631 | 0.757858283 |  |  |
| **Plasmid** | **Ct values, *YML085C* gene** | ***YML085C* gene copy number^1^** | **Copy number normalized by *YML085C* gene** | | |
| P950 | 16.3 | 3406833.475 | 1 | | |
|  | 16 | 4194304 | 1 | | |
|  | 16 | 4194304 | 1 | | |
|  | 16.1 | 3913424.009 | 1 | | |
| P1210 | 16.3 | 3406833.475 | 1 | | |
|  | 16.6 | 2767208.654 | 1 | | |
|  | 15.8 | 4817990.105 | 1 | | |
|  | 15.7 | 5163793.937 | 1 | | |
| P1038 | 15.4 | 6357376.058 | 1 | | |
|  | 15.5 | 5931641.602 | 1 | | |
|  | 15.4 | 6357376.058 | 1 | | |
|  | 15.5 | 5931641.602 | 1 | | |
| P1172 | 14.9 | 8990687.442 | 1 | | |
|  | 15.1 | 7826848.017 | 1 | | |
|  | 16.1 | 3913424.009 | 1 | | |
|  | 15.9 | 4495343.721 | 1 | | |
| Control Y1377 | 16.3 | 3406833.475 | 1 | | |
|  | 16.5 | 2965820.801 | 1 | | |
| Control Y997 | 18.7 | 645474.2422 | 1 | | |
|  | 18.4 | 794672.0072 | 1 | | |

The total DNA was isolated from two transformants of each type and the samples were run in duplicates.

^1^The gene copy number was estimated by the following formula: X = 2 ^(38-Ct)^; we assumed that Ct of 38 represents 1 copy of template in the sample to linearize the data. The *URA3* gene copy number for each strain was further normalized by *YML085C* (encoding α-tubulin).

^2^Relative DNA copy was estimated by normalizing DNA copy number of tested transformants carrying circular plasmids (P950, P1210, P1038 and P1172) to the control strain Y1377, which was estimated to carry 2 copies of *URA3* gene by Southern blotting (Fig S5).
